# Polypharmacy Management in the Older Adults: A Scoping Review of Available Interventions

**DOI:** 10.3389/fphar.2021.734045

**Published:** 2021-11-26

**Authors:** M. Kurczewska-Michalak, P. Lewek, B. Jankowska-Polańska, A. Giardini, N. Granata, M. Maffoni, E. Costa, L. Midão, P. Kardas

**Affiliations:** ^1^ Department of Family Medicine, Medical University of Lodz, Lodz, Poland; ^2^ Department of Clinical Nursing, Faculty of Health Science, Wroclaw Medical University, Wroclaw, Poland; ^3^ IT Department, Istituti Clinici Scientifici Maugeri IRCCS, Pavia, Italy; ^4^ Psychology Unit, Istituti Clinici Scientifici Maugeri IRCCS, Montescano Institute, Pavia, Italy; ^5^ UCIBIO/REQUIMTE, Faculty of Pharmacy and Porto4Ageing, University of Porto, Porto, Portugal

**Keywords:** polypharmacy, elderly, older adults, adverse drug event, adverse drug reaction, explicit criteria, inappropriate prescribing, multimorbidity

## Abstract

**Background:** Polypharmacy paves the way for non-adherence, adverse drug reactions, negative health outcomes, increased use of healthcare services and rising costs. Since it is most prevalent in the older adults, there is an urgent need for introducing effective strategies to prevent and manage the problem in this age group.

**Purpose:** To perform a scoping review critically analysing the available literature referring to the issue of polypharmacy management in the older adults and provide narrative summary.

**Data sources:** Articles published between January 2010–March 2018 indexed in CINHAL, EMBASE and PubMed addressing polypharmacy management in the older adults.

**Results:** Our search identified 49 papers. Among the identified interventions, the most often recommended ones involved various types of drug reviews based on either implicit or explicit criteria. Implicit criteria-based approaches are used infrequently due to their subjectivity, and limited implementability. Most of the publications advocate the use of explicit criteria, such as e.g. STOPP/START, Beers and Medication Appropriateness Index (MAI). However, their applicability is also limited due to long lists of potentially inappropriate medications covered. To overcome this obstacle, such instruments are often embedded in computerised clinical decision support systems.

**Conclusion:** Multiple approaches towards polypharmacy management are advised in current literature. They vary in terms of their complexity, applicability and usability, and no “gold standard” is identifiable. For practical reasons, explicit criteria-based drug reviews seem to be advisable. Having in mind that in general, polypharmacy management in the older adults is underused, both individual stakeholders, as well as policymakers should strengthen their efforts to promote these activities more strongly.

## Introduction

Recently, polypharmacy (also called polytherapy or polypragmasy) became an important public health problem due to its far-reaching consequences, such as possible negative effects on individual health, as well as increased use of healthcare services and costs (Fried et al., 2014). In particular, polypharmacy is known to cause a higher risk of adverse drug events and drug-drug interactions. It also often leads to medication non-adherence. All these provide negative health outcomes as well as increased risk of geriatric syndromes (e.g., cognitive impairment, or falls). This, in turn, leads to increased risk of hospitalization and institutionalization, as well as much greater health care expenditures (Maher et al., 2014). Therefore, polypharmacy is considered to be “one of the greatest prescribing challenges” (Payne and Avery, 2011).

Obviously, polypharmacy is not limited to older adults. Nevertheless, the highest prevalence of this scenario comes with older age. A nationwide cohort study in Sweden among individuals aged ≥65 has found prevalence of polypharmacy reaching 44%, and prevalence of extreme polypharmacy (defined as taking ten drugs or more) of 11.7% (Morin et al., 2018). Data from the United Kingdom highlight that 20.8% of individuals with two clinical conditions have been prescribed four to nine medicines, whereas 10.1% of them—ten or more medicines. In patients with six or more comorbidities, relevant values were 47.7 and 41.7%, respectively, and these figures increased with age (Barnett et al., 2012). In Poland, polypharmacy has been observed among 55.0% of the citizens aged 80+ (Kardas et al., 2021). Scottish data show that around 35% of those aged 85 years and above receive more than ten medicines (Stewart et al., 2017a). A recent analysis of a large European cohort has found polypharmacy (defined as concurrent use of five or more medications) to be present in 32.1% of citizens aged 65 years or above, ranging from 26.3 to 39.9% across the studied countries (Midão et al., 2018). High prevalence of polypharmacy in the older adults has also been observed outside Europe, e.g., in countries such as Brazil (Pereira et al., 2017) and United States (Quinn and Shah, 2017).

Thus, the burden of polypharmacy is a direct consequence of demographic challenge which, though observed worldwide, is particularly pronounced in Europe. According to Eurostat data, currently those aged 65 years or above, account for 19.2% of the European Union’s population, and this proportion is expected to rise up to 29.1% by 2080, whereas percentage of those aged over 80 years, is expected to increase even more dramatically—from the present 5.4–12.7% (Eurostat (2015). People i, 2015).

The longer citizens live, the higher are the chances of multimorbidity which is defined by the World Health Organization as “the co-occurrence of two or more chronic medical conditions in one person” (World Health Organization, 2008). Prolonged life expectancy, the privilege of people living in the 21st century, means much longer years lived with chronic conditions the number of which grows even more with age. Current statistics estimate that over 70% of people aged over 65 years are affected by multimorbidity (National Guideline Centre, 2016). It has a major impact on healthcare systems, e.g., primary care physicians in England care for patients with multimorbidity in 78% of their consultations (Salisbury et al., 2011), whilst in several other settings, e.g., geriatrics, this percentage may reach 100%.

Ageing and multimorbidity, i.e., two interlinked factors mentioned above, are to a large extent responsible for the observed rapid rise in global prevalence of polypharmacy (Guthrie et al., 2015). However, the current paradigm of healthcare seriously increases the chances of polypharmacy in the older adults as well. Undoubtedly, it is a consequence of single-disease oriented guidelines promoting pharmacotherapy as a routine solution. This approach leads to undesirable effects, such as difficulties in integrating care in multimorbidity cases, poor communication between patients, carers and their multiple care providers, and a lack of patient-focused (rather than condition-focused) care plans (Boyd et al., 2005; May et al., 2009). Unfortunately, the guidelines only seldom tend to address the complex nature of multimorbidity trying to address it from the patient’s perspective in order to prioritize certain conditions or treatments over the other ones, thus reducing the burden of prescribed drugs (Montori et al., 2013; Farmer et al., 2016). Similarly, “defensive medicine” makes the initiation of therapy easy and always correct, contrary to a more conservative approach which accepts that not every condition is automatically the reason for taking a medication, thus giving both the prescriber and the patient more freedom in making their choices based on accepted priorities (Austad et al., 2016).

Despite the significance of the problems created by polypharmacy in the older adults, this subject is only seldom tackled in European countries in a systematic way. An extensive search for polypharmacy guidance documents (both published in peer-reviewed journals and made available as grey literature) performed recently across Europe has identified only five European countries that actually have such documents targeting older patients (Stewart et al., 2017a).

There is a variety of tools aimed at reduction of inappropriate polypharmacy using either implicit (judgement-based) or explicit (item list-based) criteria (Kaufmann et al., 2014). Unfortunately, their practical implementation in older adults care is very limited. Recent research shows that healthcare professionals (HCPs) are often either unaware of such tools or disregard them as not being user-friendly (Mc Namara et al., 2017). For example, the use of various forms of drug reviews has been reported in half of 32 studied European countries only (Bulajeva et al., 2014).

Under such circumstances, healthcare professionals should be supported and motivated to implement polypharmacy targeting interventions. Therefore, the overall aim of this paper was to summarize available information on the methods to prevent and manage polypharmacy in the older adults. Accepting the perspective of practical approach and pragmatic guidance to polypharmacy management, the objective of this scoping review was to map available interventions and more complex strategies, and discuss their implementability. The rationale behind the approach was a common belief that there is no “one-size-fits-all” solution for polypharmacy management in the older adults. Therefore, in order to help HCPs select an approach that would satisfy their requirements best and increase overall application of polypharmacy management, the literature search strategy was designed to identify the scientific publications detailing a broad spectrum of interventions available for polypharmacy management in the older adults. In order to reflect the state-of-the-art findings, the literature search was limited to items published from 2010 onward.

## Materials and Methods

### Search Strategy

In this review, the Preferred Reporting Items for Systematic Reviews and Meta-Analyses (PRISMA) guidelines were followed (Moher et al., 2009). The electronic databases, i.e., CINHAL, EMBASE and PubMed, were systematically searched in accordance with the predefined literature search strategy based on a various combination of keywords including “polypharmacy” and its equivalents, terms corresponding to a systematic approach to polypharmacy management, such as “intervention” etc., and various identifiers of older age. The Supplementary Online Material S1 provides the combination of search terms that were used to identify relevant publications.

### Inclusion Criteria

Publications were included if: (A) they outlined interventions addressing polypharmacy (however, not implementation of guidelines) in the older adults in any of the following settings: 1) clinical practice, 2) health care systems, 3) scientific research; and (B) they were published in the years between 2010 and 2018. What is noteworthy is that the definition of an “intervention” was not explicit in order to allow for a broad spectrum of search results that could be of potential interest to the readers. Similarly, we accepted various definitions of the “elderly” used by the authors, not limited to the traditional convention defining the “elderly” as those aged 65 years or above. (Orimo, 2006).

### Exclusion Criteria

Articles were excluded if they: 1) were not peer-reviewed; 2) were written in a language other than English; 3) were not devoted to interventions addressing polypharmacy; or 4); did not present intervention descriptions in full details (e.g., letters, comments, conference proceedings, editorials, erratum, etc., as opposed to original articles, reviews, systematic reviews, randomized controlled trials and guidelines).

### Study Selection

Studies meeting the inclusion criteria were initially selected, based on screening the titles and abstracts by one researcher (PL). Copies of full-text papers considered as potentially relevant after the first screening were then fully analysed independently by two researchers (out of the three: BJ-P, MK-M, and PL). In the case of different opinions on possible inclusion of an article into the study, the third author (PK) was consulted to reach a consensus.

### Data Extraction Process and Analysis

The data was extracted from each eligible paper according to the predefined framework which included the source, year of publication, country of origin, type of the publication, definitions of polypharmacy used by the authors, target for intervention (i.e., multimorbidity or individual disease typical for elderly people), characteristics of intervention, settings, healthcare professionals involved in/suggested to deliver the intervention, and results of intervention implementation (for publications assessing implementation of interventions only). The extracted data are presented in the Supplementary Online Material S2. Further elaboration of the extracted data involved grouping according to the predefined criteria and a statistical analysis with descriptive statistics. The final analysis of the extracted data took the form of a narrative, descriptive summary and synthesis.

## Results

### Characteristics of Selected Studies

**FIGURE 1:**
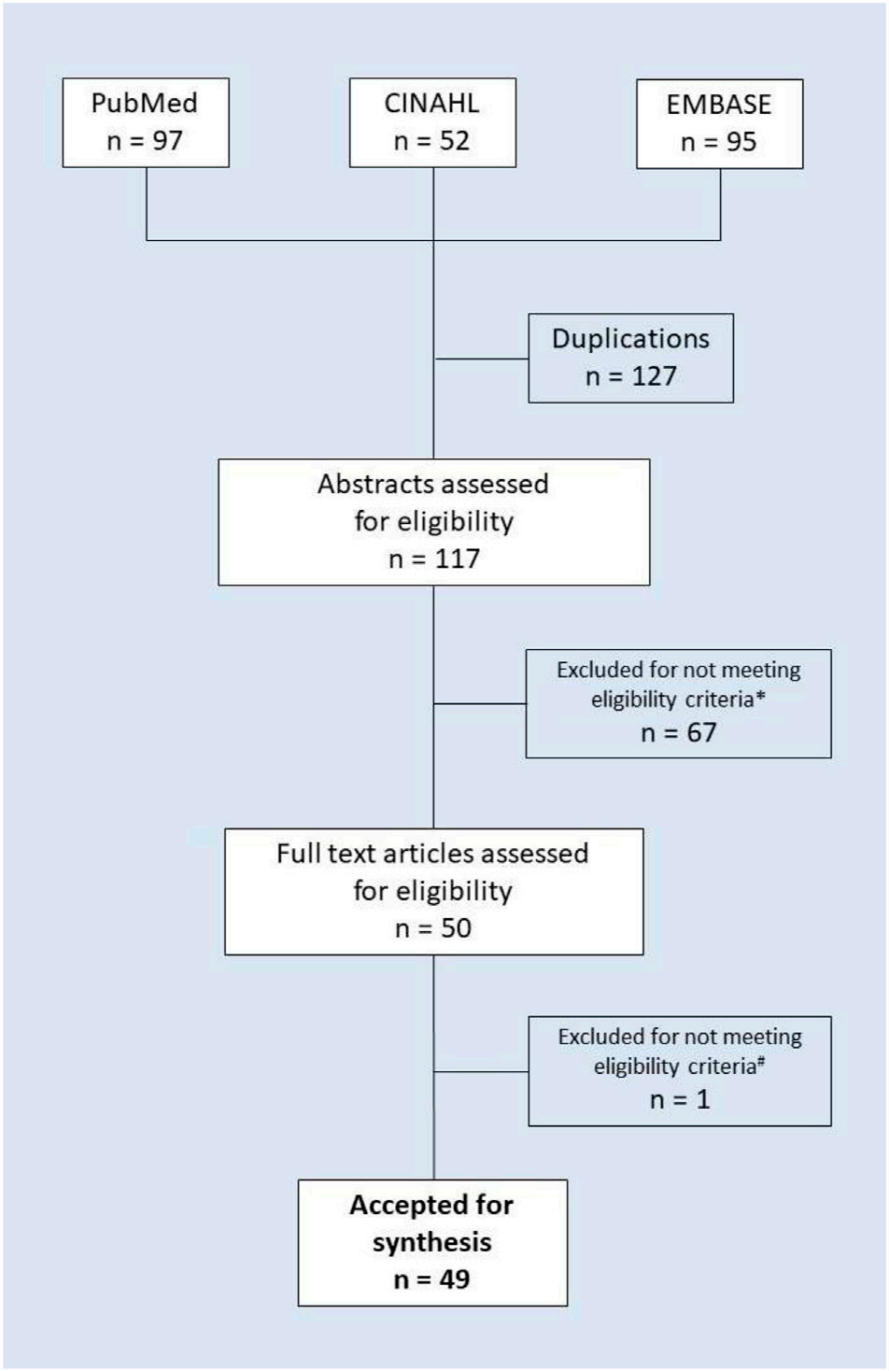
PRISMA flow chart of the literature search and study selection. Note: * Excluded due to not detailing interventions to manage polypharmacy (56 items) or not meeting other eligibility criteria (e.g., not providing the details of the intervention, 11 items in total); ^#^ excluded for not meeting eligibility criteria (non-English-language publication).

The literature search included 244 publications. Subsequently, 127 duplicates were removed, and the titles and abstracts of the remaining 117 articles were reviewed, which resulted in elimination of 67 papers that did not meet the inclusion criteria. A further detailed review of the full-text articles led to elimination of another paper. A final set of 49 articles that met the inclusion criteria was accepted for synthesis. For details of article screening and the exclusion process, see the PRISMA flow chart in Figure 1. The identified publications originated from a variety of European as well as non-European countries and included original articles, reviews, systematic reviews, randomized controlled trials and guidelines. A few papers were focused on one specific disease characteristic for older people [e.g., diabetes (Dunning, 2017), hip fracture (Komagamine and Hagane, 2017), etc.], whereas a majority of the publications did not define the type of disease. One study was focused on the patients with multimorbidity (Bokhof and Junius-Walker, 2016). All the reviewed studies were focused on elderly patients.

### Aims of Identified Interventions

Across the reviewed literature, some attention is paid to prevention of polypharmacy. Optimal or appropriate prescribing was advised as a general method of polypharmacy prevention (Kaufman, 2011; Nobili et al., 2011; Cadogan et al., 2015; Cadogan et al., 2016; Cadogan et al., 2017). This recommendation, however, was not necessarily followed by detailed practical guidance. Only one publication provides recommendations on how to prevent polypharmacy in very specific patients, i.e., critically ill older adults who, when staying at an intensive care unit, are at risk of developing delirium (Garpestad and Devlin, 2017). In fact, strategies of polypharmacy management identified in our search predominantly target correction of polypharmacy. Specific aims of relevant interventions include one or several out of the below-listed ones:

1. Reduction of polypharmacy (lowering the number of drugs prescribed and/or used)
2. Increasing the use of a recommended medication
3. Lowering the costs (drug costs, and/or overall healthcare system expenditures)
4. Enhancing patient adherence to medication
5. Increasing effectiveness of drug therapy (e.g., avoidance of hospitalisations, etc.)
6. Securing patient safety (e.g., avoidance of adverse drug reactions)

### Targets of Identified Interventions

Although the role of patients is emphasized, and relevant recommendations include better patients’ health literacy and awareness of their complex multiple medication regimens (Bokhof and Junius-Walker, 2016), patients are not perceived as those who actively initiate any formalised action against polypharmacy. In fact, it is also suggested that general practitioners (GPs) might support patients by “inviting” their contribution to polypharmacy and medication safety, as their awareness of the significance of their active role in addressing polypharmacy is currently very low (Schöpf et al., 2017).

Thus, the reviewed literature supports the use of interventions targeting polypharmacy which are initiated by healthcare professionals. A suggested trigger to employ such an intervention is just presence of polypharmacy in an older adult. This advice, however, is not easy to implement due to current lack of common standard definition of polypharmacy. In fact, the authors adopted various existing definitions, as illustrated in Table 1. Among them, the most commonly used definition of polypharmacy was taking concurrently five or more medications. However, in some publications other threshold values were also used, ranging from 1 to >9. Moreover, in several papers a qualitative approach to polypharmacy definition was preferred and the most common one was the imprecise definition describing it as “the use of a number of different medicines possibly prescribed by different doctors and often filled in different pharmacies, by a patient who may have one or several health problems” (Kaufman, 2011; Nobili et al., 2011; Clyne et al., 2016; Dunning, 2017; Lin et al., 2018). Finally, in nine papers the operational definition of polypharmacy was not precisely detailed (Planton and Edlund, 2010; Sergi et al., 2011; Mansur et al., 2012; Van der Linden et al., 2014; Yamanouchi et al., 2014; Cadogan et al., 2016; Garpestad and Devlin, 2017; Heaton et al., 2017; McNicholl et al., 2017) leaving it open to individual interpretation.

**TABLE 1:** Definition of polypharmacy used in reviewed publications.

Definition of polypharmacy			
Type of definition	—	—	References
Numerical	Number of medications	Number of studies	—
	1	1	Bokhof and Junius-Walker, (2016)
	>3	1	Zelko et al. (2016)
	≥4	7	Kaufman. (2011); Patterson et al. (2012); Patterson et al. (2014); Cooper et al. (2015); Stewart et al. (2017b); Cadogan et al. (2017); Patton et al. (2017)
	>5	2	Doan et al. (2013); Kim and Parish. (2017)
	≥5	17	Clyne et al. (2012); Eyigor and Kutsal (2012); Sabzwari et al. (2013); Bergert et al. (2014); Jódar-Sánchez et al. (2015); Kann et al. (2015); Chau et al. (2016); Sharma et al. (2016); Sinnige et al. (2016); Urfer et al. (2016); Franco et al. (2017); Kaufman. (2017); Komagamine and Hagane. (2017); Malet-Larrea et al. (2017); Schöpf et al. (2017); Tommelein et al. (2017); Lin et al. (2018)
	5–9	1	Harugeri et al. (2010)
	≥9	2	(Kojima et al. (2014); Jokanovic et al. (2017))

Qualitative	Definition	Number of studies	References
	1. The use of a number of different medicines possibly prescribed by different doctors and often filled in different pharmacies, by a patient who may have one or several health problems	5	Kaufman. (2011); Nobili et al. (2011); Clyne et al. (2016); Dunning. (2017); Lin et al. (2018)
	2. The use of multiple medicines and/or more medicines than clinically indicated	4	Patterson et al. (2014); Wilson et al. (2015); Rodrigues and Oliveira. (2016); Levy. (2017)
	3. Prescribing of multiple medicines (this includes “inappropriate polypharmacy” and “appropriate polypharmacy”)	1	Stewart et al. (2017a)
	4. At risk of inappropriate prescribing and adverse drug events	1	Hughes et al. (2016)

### Who Should Provide a Polypharmacy Management Intervention

The reviewed literature pointed to a range of healthcare professionals who may or should provide polypharmacy addressing intervention. The most common setting in which polypharmacy management interventions were most successful was primary care and they were implemented either by GPs, or by primary healthcare team (Kaufman, 2011; Nobili et al., 2011; Sabzwari et al., 2013; Bergert et al., 2014; Kann et al., 2015; Bokhof and Junius-Walker, 2016; Cadogan et al., 2016; Clyne et al., 2016; Sinnige et al., 2016; Cadogan et al., 2017; Franco et al., 2017; Schöpf et al., 2017; Tommelein et al., 2017). However, some interventions were provided at community or hospital pharmacies, by pharmacists alone, in the form of pharmaceutical care, or in cooperation with a physician, e.g., under an umbrella of collaborative physician-pharmacist medication therapy management (MTM) program (Mansur et al., 2012; Patterson et al., 2012; Doan et al., 2013; Cooper et al., 2015; Jódar-Sánchez et al., 2015; Wilson et al., 2015; Cadogan et al., 2016; Chau et al., 2016; Jokanovic et al., 2017; Komagamine and Hagane, 2017; Malet-Larrea et al., 2017; McNicholl et al., 2017; McNicholl et al., 2017; Tommelein et al., 2017; Lin et al., 2018). Specialists who are perfectly prepared to take care of polypharmacy in the older adults are geriatricians, thus relevant interventions could be included in the geriatric consultation (Eyigor and Kutsal, 2012; Kojima et al., 2014; Van der Linden et al., 2014). Finally, other settings also allow for polypharmacy interventions which have been successfully provided in various hospital settings such as teaching hospitals (Harugeri et al., 2010; Urfer et al., 2016; Lin et al., 2018), acute care hospitals (Komagamine and Hagane, 2017), acute geriatric wards (Mansur et al., 2012; Van der Linden et al., 2014). It is worth emphasizing that such interventions are also advisable in the case of residential aged care facilities (Kojima et al., 2014; Jokanovic et al., 2017). Some studies highlight the need for an interdisciplinary approach, e.g., in order to execute Comprehensive Geriatric Assessment (CGA), the authors suggest an interdisciplinary team comprising nurses, occupational and physical therapists, social workers, general practitioners and geriatricians (Sergi et al., 2011).

### How Often Should an Intervention Be Provided

The available literature does not pay much attention to the question of how often interventions targeting polypharmacy should be repeated. According to one publication included in our review, GPs should scrutinize senior people’s medications on each consultation whenever a patient meets the criteria of polypharmacy (Dunning, 2017). The recently published WHO report on medication safety in polypharmacy generally recommends that “appropriate polypharmacy should be considered at every point of initiation of a new treatment for the patient, and when the patient moves across different health care settings.” (World Health Organization, 2019) As for residents of care homes, the NICE guidelines advise that an interval in medication reviews “should be no more than 1 year” and that many residents may require reviews more often. (National Institute for Health and Clinical Excellence, 2015) Obviously, practical implementation of relevant interventions is limited by many factors, such as the availability of qualified staff, a paradigm of the local healthcare system, reimbursement of the intervention, etc.

### Details of Identified Interventions

Full list of all types of interventions identified in the reviewed studies is presented in Table 2 **.**

**TABLE 2:** Polypharmacy interventions identified in reviewed publications.

Intervention		Number of publications	References
Optimal/appropriate prescribing		5	Kaufman (2011); Nobili et al. (2011); Cadogan et al. (2015); Cadogan et al. (2016); Cadogan et al. (2017)
Deprescribing		7	Bokhof and Junius-Walker (2016); Sharma et al. (2016); Urfer et al. (2016); Jokanovic et al. (2017); Kaufman (2017); Komagamine and Hagane (2017); Schöpf et al. (2017)
Drug review		18	Planton and Edlund (2010); Kaufman, (2011); Nobili et al. (2011); Sergi et al. (2011); Kojima et al. (2014); Wilson et al. (2015); Chau et al. (2016); Hughes et al. (2016); Sharma et al. (2016); Urfer et al. (2016); Stewart et al. (2017b); Cadogan et al. (2017); Dunning (2017); Jokanovic et al. (2017); Kaufman (2017); Komagamine and Hagane (2017); Levy (2017); McNicholl et al. (2017)
Medication review with follow-up (MRF)		2	Jódar-Sánchez et al. (2015); Malet-Larrea et al. (2017)
Comprehensive program of polypharmacy management		1	Kaufman, (2017)
Pharmaceutical care		3	Patterson et al. (2012); Cooper et al. (2015); Tommelein et al. (2017)
Collaborative physician—pharmacist medication therapy management (MTM)		1	Lin et al. (2018)
Comprehensive Geriatric Assessment		4	Sergi et al. (2011); Eyigor and Kutsal (2012); Sharma et al. (2016); Pazan and Wehling (2017)
Validated screening tools
	STOPP/START	19	Nobili et al. (2011); Sergi et al. (2011); Eyigor and Kutsal (2012); Patterson et al. (2012); Bergert et al. (2014); Patterson et al. (2014); Cooper et al. (2015); Chau et al. (2016); Clyne et al. (2016); Hughes et al. (2016); Rodrigues and Oliveira (2016); Sharma et al. (2016); Urfer et al. (2016); Cadogan et al. (2017); Franco et al. (2017); Kim and Parish (2017); Komagamine and Hagane (2017); Levy (2017); McNicholl et al. (2017)
	Beers criteria	17	Planton and Edlund (2010); Nobili et al. (2011); Sergi et al. (2011); Eyigor and Kutsal (2012); Patterson et al. (2012); Sabzwari et al. (2013); Kojima et al. (2014); Patterson et al. (2014); Cooper et al. (2015); Clyne et al. (2016); Hughes et al. (2016); Rodrigues and Oliveira (2016); Sharma et al. (2016); Kim and Parish (2017); Komagamine and Hagane (2017); Levy (2017); McNicholl et al. (2017)
	MAI	11	Sergi et al. (2011); Barnett et al. (2012); Eyigor and Kutsal (2012); Patterson et al. (2012); Bergert et al. (2014); Patterson et al. (2014); Cooper et al. (2015); Rodrigues and Oliveira (2016); Sharma et al. (2016); Cadogan et al. (2017); Patton et al. (2017)
	NORGEP	3	Nobili et al. (2011); Hughes et al. (2016); Rodrigues and Oliveira (2016)
	IPET	1	Eyigor and Kutsal, (2012)
	McLeod	4	Nobili et al. (2011); Patterson et al. (2012); Patterson et al. (2014); Cooper et al. (2015)
	PIM	5	Nobili et al. (2011); Kojima et al. (2014); Van der Linden et al. (2014); Sharma et al. (2016); Levy (2017)
	PIP	5	Stewart et al. (2017b); Franco et al. (2017); Kaufman (2017); McNicholl et al. (2017); Tommelein et al. (2017)
	PRISCUS	2	Bergert et al. (2014); Hughes et al. (2016)
	MRCI	2	Mansur et al. (2012); Cadogan et al. (2017)
	ARMOR	2	Planton and Edlund (2010); Levy (2017)
New screening tool	
	RASP 2.0	1	Van der Linden et al. (2014)
	GheOPS tool	1	Tommelein et al. (2017)
	multidrug cytochrome-specific software program	1	Doan et al. (2013)
Computerised decision support		6	Eyigor and Kutsal (2012); Patterson et al. (2012); Patterson et al. (2014); Cooper et al. (2015); Bokhof and Junius-Walker (2016); Sinnige et al. (2016)

Note: STOPP–Screening Tool of Older Persons’ Potentially Inappropriate Prescriptions; START–Screening Tool to alert Doctors to the Right Treatment; MAI–Medication Appropriateness Index; IPET–Inappropriate Prescribing in the Elderly Tool; NORGEP–The Norwegian General Practice criteria; McLeod–McLeod criteria; PIM–Potentially Inappropriate Medication; PIP–Potentially Inappropriate Prescribing; PIM–Potentially Inappropriate Medications; EMR–Electronic Medical Record; MRCI–Medication Regimen Complexity Index; PRISCUS–PhaRmaCotheRaPy In eldeRly PatIentS; ARMOR–Assess, Review, Minimize, Optimize, Reassess.

For obvious reasons, effective management of polypharmacy should start with its prevention. Appropriate prescribing is the method that undoubtedly satisfies this expectation. Thus, a thorough risk–benefit analysis of each medicine should be made whenever any drug is prescribed (Kaufman, 2011; Nobili et al., 2011; Bokhof and Junius-Walker, 2016; Cadogan et al., 2016; Cadogan et al., 2017)^.^ If, however, polypharmacy is already in place, deprescribing is another logical step to be taken, as suggested by several publications (Bokhof and Junius-Walker, 2016; Sharma et al., 2016; Urfer et al., 2016; Jokanovic et al., 2017; Kaufman, 2017; Komagamine and Hagane, 2017; Schöpf et al., 2017). Although not limited to, the concept, aims, and practice of deprescribing overlap much with polypharmacy management. One of its definitions describes it as “the process of withdrawal of an inappropriate medication, supervised by a health professional with the goal of managing polypharmacy and improving outcomes” (Reeve et al., 2015). This broad concept has been supported by specific guidance, e.g., patient-centred deprescribing strategy, proposed in one of the publications (Kaufman, 2017). The strategy includes five steps: 1. comprehensive medication history; 2. identification of potentially inappropriate medications; 3. determination if medication can be ceased and prioritisation; 4. planning and executing withdrawal; and finally, 5. monitoring, support and documentation.

A practical implementation of the deprescribing process in older adults may be guided by four crucial questions as proposed by Page et al. (2016), i.e.:

1. Is it an inappropriate prescription (e.g., a case without clear indication, obvious contraindications, or a consequence of “prescribing cascade”)?
2. Does the drug lead to adverse effects or interactions that outweigh symptomatic effects or potential future benefits?
3. Are drugs taken for symptom relief but the symptoms are stable?
4. Is drug intended to prevent serious future events but the potential benefit is unlikely to be realised due to limited life expectancy?

If the answer to any of these questions is positive, then the medication should be considered for deprescribing.

No matter whether deprescribing comes under its own name, or not, it is the major aim of corrective polypharmacy addressing interventions. Perhaps, the most well-known and crucial part of this process is a drug review.

Indeed, various forms of drug reviews and identification of potentially inappropriate medications were the most often suggested procedures according to our literature review (see Table 2). Drug reviews might be stand-alone procedures. However, they might be also embedded in more complex programs, being the core item of e.g., Comprehensive Geriatric Assessment (Sergi et al., 2011; Eyigor and Kutsal, 2012; Sharma et al., 2016; Pazan and Wehling, 2017), pharmaceutical care (Patterson et al., 2012; Cooper et al., 2015; Tommelein et al., 2017), and collaborative physician—pharmacist medication therapy management (Lin et al., 2018).

Effective polypharmacy management with drugs reviews may require that several additional factors are taken into consideration, such as:

• Settings: hospital vs. outpatient, in the latter case: primary care vs. specialised care (e.g., outpatient geriatric clinic).
• A healthcare professional to perform drug review (e.g., a physician, pharmacist, nurse, other)
• The purpose and related scope of the drug review
• Criteria to guide drug review (implicit vs. explicit)
• A tool to base drug review on (comprehensive vs. limited in scope; validated vs. non-validated)
• A method used for drug review (manual vs. supported by a computerised clinical decision system)

Depending on their purpose, drug reviews may have a different scope. Therefore, current literature distinguishes three types of such reviews (Shaw and Seal, 2015; Clyne et al., 2008):

• Type 1—Prescription review, performed often without the patient, addressing technical issues relating to the prescription (e.g., duplications, possible drug-drug interactions etc.)
• Type 2—Concordance and compliance review, performed most often in the patient’s presence, addressing issues relating to their medicine-taking behaviour
• Type 3—Clinical medication review, requiring the patient’s presence, addressing issues relating to their use of medicines in the context of their clinical conditions

Drug reviews are advised to be undertaken by all physicians and particularly frequently by GPs (Kaufman, 2011). Pharmacists seem to be competent to carry out drug reviews as well. The medication review with follow-up (MRF) performed by pharmacists in community pharmacies provided a decreased number of prescribed medicines, reduction of emergency department visits and hospitalizations, improvement of quality of life of patients, and it also lowered the mean daily cost of prescribed medication (Jódar-Sánchez et al., 2015; Malet-Larrea et al., 2017). In Spanish study, the cost analysis showed that MRF saved the national health system € 97 per patient in 6 months. It was calculated that for every 1 euro invested in MRF a service returned a benefit of € 3.3 to € 6.2 (Malet-Larrea et al., 2017)^.^

In practical terms, drug reviews are usually formalised, and driven by either implicit (judgement-based), or explicit criteria. Due to their usefulness, explicit criteria-based screening tools are used most often to help systematic assessment of drug safety and appropriateness. In publications covered by this review, the tools most often recommended for use in clinical practice were the ones based on such criteria, i.e., STOPP/START criteria, Beers Criteria and MAI index. A short overview of these three instruments is presented below.

### Beers Criteria

In 1991, a geriatrician Mark H. Beers published criteria on potentially inappropriate use of medication in the older adults agreed by experts (Beers, 1997). After a few updates, the last version in 2019 (stewarded by the American Geriatrics Society) included not only evidence-based recommendations on drugs to be avoided, but also guidance on which medication should be used with caution, expected to cause significant drug-drug interactions or be reduced depending on the kidney function in seniors. (By the 2019 American Geri, 2019) These are the longest running explicit criteria for potentially inappropriate medication for older patients with five updates since the first publication. They are useful as a clinical, educational and public health tool developed to be used in conjunction with healthcare providers. However, the main disadvantage of Beers criteria is the fact that two large European studies have shown a lack of their association with adverse drug reactions (Onder et al., 2005; Laroche et al., 2007). Due to a large number of presented drugs, it is a challenge to create a simple checklist using these criteria. Also, additional software is required to take full advantage of its potential (Levy, 2017). It should be emphasized that being of American origin, Beers criteria may include or miss medications used or not in Europe (O’Mahony, 2019).

### STOPP/START Criteria

Proposed for the first time in 2008 by an Irish geriatrician Denis O’Mahony and his colleagues, it is a list of potential prescribing omissions (underprescribed drugs) and potentially inappropriate medications for seniors. In its second version published in 2015, the list included revised criteria included in the first version divided into groups depending on the body systems approved by 19 experts from 13 European countries^.^ (O’Mahony et al., 2015). Its definite advantage is the evidence for correlation with reduction of adverse drug events^.^ (Hamilton et al., 2011). They are endorsed and used by several European societies including the National Institute for Clinical Excellence (NICE) and the United Kingdom Royal College of General Practitioners (O’Mahony, 2019). However, these criteria (currently planned for 5-year periods) (O’Mahony, 2019) need updating, and just like other explicit criteria (e.g., Beers) they cannot evaluate drug therapy omission, adherence, life expectancy, issues related to comorbidities or patient preferences. Some studies show that they ignore a majority of drug-related problems in seniors (Verdoorn et al., 2015).

### Medication Appropriateness Index

In 1992, a clinical pharmacist Joseph Hanlon and a geriatrician Kenneth Schmader proposed criteria in a form of ten questions enabling assessment of drugs taken by a patient. (Hanlon et al., 1992) By providing an answer to each question based on a three-point scale (“A” being appropriate, “B” being marginally appropriate, and “C” being inappropriate), appropriateness index can be calculated for each drug. A weighting system for each MAI question has also been developed. In order to obtain a total MAI score per person, the scores for individual drugs were summed up (Hanlon et al., 1992). This method was quite easy to perform; therefore, it was employed in multiple studies. It also considered drug-drug or drug-disease interactions. However, its main disadvantage was the time needed for answering the questions. It took 10 minutes per drug, which (Hanlon et al., 1992) made it impossible to use MAI in a busy outpatient clinic without application of computer software. Moreover, patient medication adherence was not included. The MAI score did not help the clinician to prioritize which drugs should be changed, neither did it provide assistance in how to modify drug regimens to avoid adverse drug withdrawal events that could occur in older adults. (Hanlon and Schmader, 2013).

Along with the validated reliable instruments, we have identified three studies based on the development and/or testing of new screening tools (Doan et al., 2013; Van der Linden et al., 2014; Tommelein et al., 2017). One of them was focused on development and validation of RASP checklist to systematically identify Potentially Inappropriate Medications (PIMs) in the older adults (Van der Linden et al., 2014). The second study used GheOP³S tool for identification of potentially inappropriate prescribing (PIP) in community-dwelling older people on polypharmacy (Tommelein et al., 2017). The third one analysed CYP-mediated patients’ drug-drug interactions (Doan et al., 2013). Detailed characteristics of these studies are provided in the Supplementary Online Material S3.

Implicit criteria-based approaches are usually employed by more complex strategies, such as comprehensive geriatric assessment (CGA). Typically, CGA includes a drug review, performed with the involvement of interdisciplinary team comprising nurses, occupational and physical therapists, social workers, general practitioners and geriatricians (Sergi et al., 2011). With the use of several evaluation tools exploring cognitive, clinical, nutritional, functional and social parameters, the team conducts a global assessment of an older adult with the primary aim of drug therapy optimisation and correction of medications used for untreated or under-treated conditions (Sergi et al., 2011).

It is noteworthy that some publications advised concurrent use of more than one screening tool. For example, one review (Planton and Edlund, 2010) suggested the use of both ARMOR (Assess, Review, Minimize, Optimize, Reassess) and Beers criteria, along with the recommendation to avoid drugs covering side effects of other drugs (i.e., the so-called “prescribing cascade”), whereas another one suggested the use of two explicit-based approaches, i.e., Beers and STOPP criteria (Levy, 2017).

Drug reviews can be further facilitated by implementing specific computerised decision support systems and mobile applications which most often use one or many validated screening tools, at first those based on explicit criteria. Such an approach proved to be an effective element of primary care and pharmaceutical care, leading to reductions in inappropriate prescribing (Patterson et al., 2012; Cooper et al., 2015). Multidimensional geriatric assessment could be also improved by dedicated IT solutions providing on-line access to information on patients, alerts indicating inappropriate drugs prescribed, assessment of the effects of accompanying diseases, reviewing potential drug-drug interactions, etc. (Eyigor and Kutsal, 2012).

### Comprehensive Strategies

Our search revealed comprehensive strategies described in dedicated guidelines. One of these, focused on geriatric patients on multimedication (Bergert et al., 2014), was designed especially for GPs. They identified eight key steps as components of appropriate prescription process:

Step 1. Patient evaluation and collecting information
Step 2. Medication review
Step 3. Agreeing with patients on treatment objectives
Step 4. Prescription decision
Step 5. Communication and obtaining patient agreement
Step 6. Drug dispensing
Step 7. Medication usage
Step 8. Monitoring and assessment

As for medication review in Step 2, these guidelines suggest the use of several instruments, including MAI, STOPP/START and PRISCUS. It is noteworthy that, in Step 3, after agreeing overall objectives of the treatment with the patient, along with their expectations for a pharmaceutical treatment, a GP is supposed to prescribe a drug (Step 4), communicate this to the patient, and obtain their agreement (Step 5).

Being one of only very few well-organized polypharmacy management programs in Europe (Stewart et al., 2017a), the NHS Scotland Polypharmacy Guidance (Wilson et al., 2015) offers probably the most complete guidance to polypharmacy management, as evaluated by our search. This guidance accepts a patient-centred approach to ensuring safe and appropriate use of medicines in polypharmacy. Therefore, it advocates a drug review process that should be focused on the patient as a whole rather than a jigsaw of conditions. The updated third edition of the guidance, published in 2018, provides a holistic model of care based on a comprehensive approach to medication review and provides healthcare professionals with practical tips to improve prescribing in polypharmacy and make it less problematic (Scottish Government Polyp, 2018). This approach may be easily adopted to the need of polypharmacy management in the older adults (Wilson et al., 2015). It recommends that clinicians step back from the usual process of chronic condition management to specifically consider the challenges of multimorbidity. They should realize that patients need a “multimorbidity focus” and initiate a process that enables patients to prioritize their own care needs.

In practical terms, the guidance is composed of seven steps to follow (see Table 3). It starts with establishing treatment objectives in cooperation with the patient (Step 1), and it is followed by identification of essential (Step 2) and unnecessary drugs (Step 3). Then, it is checked whether therapeutic objectives have been achieved (Step 4), which is followed by identification of potential or actual adverse drug reactions (Step 5). At the end of the process it is verified whether therapy costs can be minimized (Step 6) and checked if the patient is willing and able to receive drug therapy as planned (Step 7). This model provides a cohesive structure for a polypharmacy management process that is holistic, patient-centred and applicable to older adults across a range of health care settings. It should be emphasized that this model is not based on any specific explicit criteria-based tools. Instead, it uses its own set of potentially unnecessary drugs.

**TABLE 3:** An overview of key considerations of 7 Steps of NHS Scotland Polypharmacy Guidance, 3rd edition [from (Wilson et al., 2015), with modifications].

Domain	Steps	Process
Aims	1. Identify objectives of drug therapy	Review diagnoses and identify therapeutic objectives with respect to
		• Management of existing health problems
		• Prevention of future health problems
Need	2. Identify essential drug therapy	Identify essential drugs (not to be stopped without specialist advice)
		• Drugs that have essential replacement functions (e.g., thyroxine)
		• Drugs to prevent rapid symptomatic decline (e.g., drugs for Parkinson’s disease, heart failure)
	3. Does the patient take unnecessary drug therapy	Identify and review the (continued) need for drugs
		• with temporary indications
		• with higher than usual maintenance doses
		• with limited benefit in general or the indication they are used for
		• with limited benefit in the patient under review
Effectiveness	4. Are therapeutic objectives being achieved?	Identify the need for adding/intensifying drug therapy in order to achieve therapeutic objectives
		• to achieve symptom control
		• to achieve biochemical/clinical targets
		• to prevent disease progression/exacerbation
Safety	5. Does the patient have adverse drug reactions or is at risk of adverse drug reactions?	Identify patient safety risks by checking for
		• drug-disease interactions
		• drug-drug interactions
		• robustness of monitoring mechanisms for high-risk drugs and for high-risk
		drug-drug and drug-disease interactions
		• risk of accidental overdosing
		Identify adverse drug effects by checking for
		• specific symptoms/laboratory markers
		• cumulative adverse drug effects
		• drugs that may be used to treat ADRs caused by other drugs
Costeffectiveness	6. Is drug therapy costeffective?	Identify unnecessarily costly drug therapy by
		• Considering more cost-effective alternatives (but balance against effectiveness, safety, convenience)
Adherence/Patientcenteredness	7. Is the patient willing and able to take drug therapy as intended?	Identify risks to patient non-adherence by considering
		• Is the medicine in a form that the patient can take?
		• Is the dosing schedule convenient?
		• Is the patient able to take medicines as intended?
		• Is the patient’s pharmacist informed of changes to regimen?
		Ensure drug therapy changes are tailored to patient preferences by
		• Discuss with the patient/carer/or welfare proxy therapeutic objectives and treatment priorities
		• Decide with the patient/carer/or welfare proxies what medicines have an effect of sufficient magnitude to consider continuation/discontinuation

This approach is well-designed and based on strong evidence, however, it is also time—consuming. List of medications that should be considered by healthcare professionals following Steps from 2 to 7 includes almost 100 drugs, groups of drugs and scenarios. This might be a serious disadvantage, especially in primary care settings. Busy practitioners may not necessarily be able to manage that big load of data. To overcome this limitation, in Scotland, since 2013 pharmacists have been funded to work in general practice and support appropriate polypharmacy management (Mair et al., 2019). Recently, an application has also been made available for clinicians to help practical realization of this process, along with a toolkit for patients taking multiple medicines, as well as their carers to support self-management and shared decision-making during consultation and medicine reviews (The Scottish Government Polypharmacy, 2018).

It is noteworthy that from the interventions described above, several ones were analysed and checked in order to confirm their effectiveness in clinical outcomes in randomized controlled trials, interventional or prospective studies. They included several interventions, e.g., assessment of appropriateness of polypharmacy (Komagamine and Hagane, 2017; Lin et al., 2018), drug reviews (Jódar-Sánchez et al., 2015; Malet-Larrea et al., 2017; McNicholl et al., 2017) or checklists improving quality of drug prescription (Urfer et al., 2016). A complex intervention to be used in a nursing home (covering a drug list review, identification of potentially inappropriate medications using the Beers criteria, potential drug-drug interactions and contraindicated medications using the Epocrates online drug-drug interaction program) has been assessed in a prospective study which demonstrated a decrease in potentially inappropriate medications, contraindicated drugs, and medication costs. (Kojima et al., 2014) Characteristics of the studies providing evidence of effectiveness for selected interventions that have been identified in our search are presented in the Supplementary Online Material S4.

## Discussion

Our review clearly shows that current scientific literature devotes a lot of attention to polypharmacy, not only in its general aspect, but particularly focusing on older adults. Consequently, various potentially useful approaches to polypharmacy management have been described, ranging from narrow-focused screening tools up to comprehensive programs and complex strategies. This large variety of solutions enables healthcare professionals to adopt polypharmacy-addressing interventions that suit their needs and preferences, taking into account specificity of the clinical scenario. On the other hand, it may lead to obvious confusion in less experienced medical staff who, in their busy daily practice, may not find enough time or motivation to learn and implement a new service which might be certainly time-consuming. Indeed, there is evidence that the uptake of available strategies is more than limited (Mc Namara et al., 2017).

Theoretically, the most effective polypharmacy strategy could be appropriate prescribing. If each and every drug initiated in a patient satisfied the criteria of appropriate prescribing, the multidrug therapies could be avoided, and the prevalence of polypharmacy would reduce. Unfortunately, the current fragmented architecture of the healthcare systems, and single disease-oriented clinical guidelines do not help practical implementation of this concept (Farmer et al., 2016). Instruments designed to promote appropriate prescribing are mostly based on implicit criteria and thus not easy to implement, particularly in the digital version.

A very interesting finding of our review was that current literature does not perceive the patients as those who take care of their therapies in terms of initiating activities aimed at reduction of inappropriate polypharmacy. Apart from the NHS Scotland Polypharmacy Guidance, which takes the patient’s perspective into account along the whole cycle of polypharmacy management, most of other publications reserve a much less important role for the patient making them an object rather than a subject of relevant interventions. In the light of current limited use of available tools by healthcare professionals, this paradigm perhaps needs to be changed. Being provided with necessary knowledge, even an older adult may be an important ally for HCPs in adoption of polypharmacy management interventions.

In absence of patients’ pressure to get involved in polypharmacy issues, healthcare professionals are expected to self-initiate relevant activities. Here again, available literature does not help much, not providing a clear message on when to consider such an activity, and how often to include it in routine care. Perhaps, the most frustrating problem is current lack of uniform definition of polypharmacy, which not only hinders implementation of available interventions, but also makes their benchmarking much more elusive (Masnoon et al., 2017).

The most common operational definitions of polypharmacy, applied in the reviewed publications, were based on the number of concurrently prescribed and/or used drugs, with five and more being the most frequent option. This, however, deserves a comment. Although polypharmacy has numerous negative consequences, in some cases is desirable. Perhaps, for every patient there is an optimal number of drugs to be used (e.g., for hypertension to be controlled according to certain recommended levels, often two or more medications are required). It results from a rational compromise between the benefits of providing evidence-based therapies for particular conditions, and the negative consequences of using too many drugs at the same time. Thus, “appropriate polypharmacy” or “optimal polypharmacy” should be distinguished from inappropriate one (Rankin et al., 2018). Unfortunately, this distinction is subject to case-by-case approach. Therefore, it may cause confusion, as it cannot be concluded with a simple uniform threshold that would be suitable for everyone, which dichotomizes the number of drugs used concurrently to be either acceptable or too high (Masnoon et al., 2017).

Our findings undoubtedly show that available interventions might be successfully implemented by a range of healthcare professionals, first of all GPs, pharmacists, and geriatricians. Some tools are dedicated or are most suitable for each out of these groups [e.g., recommendations for treating adult and geriatric patients on multimedication designed by and for GPs (Bergert et al., 2014)], whereas others are much more generic, and might be implemented across different settings [e.g. STOPP/START (O’Mahony et al., 2015)].

Our results show that various forms of drug reviews are particularly often used for polypharmacy management in the older adults. However, despite an obvious value of drug reviews, they are not necessarily employed routinely in clinical practice. On the contrary, in Europe, various forms of these reviews were reported in only 16 out of 32 studied countries (Bulajeva et al., 2014). Most often, medication reviews were reported to be carried out in hospital settings (14 countries), followed by 13 countries reporting implementation of such a procedure in community settings, and only six in nursing homes. In community settings, those were mostly reviews targeting prescription and verifications of patients’ medicine-taking behaviours (reported in nine and 11 countries, respectively), and much less often, medication reviews in the context of patients’ clinical conditions (reported in six countries only). Another important question is which approach to choose to guide the drug review. A systematic review of tools to assess potentially inappropriate prescribing found that out of 46 different instruments identified, 39 did not have any validation in clinical settings (Kaufmann et al., 2014).

From a practical point of view, the core assumption on a strategy used for drug review is very important. According to the applied criteria, approaches may be divided into two different categories, i.e., those based on implicit and explicit criteria. Strategies based on implicit criteria involve highly individualized clinicians’ assessments relying mostly on their experience. These strategies are designed usually as protocols, algorithms or concepts examples of which are ARMOR (Haque, 2009) or the Prescribing Optimization Method (Drenth-van Maanen et al., 2009). Implicit criteria are usually short and concise. However, since they depend on clinicians’ knowledge and experience, they are highly subjective and thus, of limited applicability across patient populations, or in benchmarking (Levy, 2017). Last but not least, implementation of these strategies is very limited by the fact that they are extremely time-consuming. For example, comprehensive geriatric assessment has proved effective in reducing the number of prescriptions and daily drug doses (Sergi et al., 2011). On the other hand, it takes a lot of time, particularly when performed face-to-face with the patient (Martin-Khan et al., 2016). For all these reasons, this approach is not often used in clinical practice.

The other type of strategies aimed at reducing polypharmacy is based on explicit criteria. These are much easier to use, straightforward criteria which allow for objective elimination of inappropriate drugs, consisting mostly of lists of medications to be excluded from a patient’s treatment regimen. Most well-known examples of such an approach illustrated by our review are Beers (By the 2019 American Geri, 2019) and STOPP/START criteria (O’Mahony et al., 2015). It is noteworthy that explicit criteria are those which can be particularly well embedded in computer decision support systems and relevant applications. Interestingly, our findings show that explicit STOPP/START and Beers criteria are the validated tools most often used in polypharmacy management in the older adults. However, even these criteria are not generally accepted as a “golden standard”. On the contrary, they are criticized for not listing a relevant number of drug-related problems (Verdoorn et al., 2015) and a limited clinical value (Parekh et al., 2019). Some authors suggest that they should be used in a complementary fashion to improve detection of adverse drug reactions (Brown et al., 2016). Actually, some decision support systems use both these sets of criteria in parallel (Monteiro et al., 20192019). Moreover, practical use of these criteria might be difficult. A recent systematic review on identifying potentially inappropriate prescribing in older people with dementia found that out of 15 studies using the Beers criteria, as many as 13 did not use the full tool (Hukins et al., 2019). Due to the large number of potentially contraindicated medications listed (114 recommendations in the START/STOPP and 90 in the Beers), the use of these criteria is particularly limited in primary care (Croke, 2020).

Complex and time-consuming nature of polypharmacy management encourages the use of various decision-support systems. Indeed, a rising number of computer decision-support systems and dedicated applications is available to help clinicians manage polytherapy in real life conditions of busy practice (Eyigor and Kutsal, 2012; Patterson et al., 2012; Patterson et al., 2014; Cooper et al., 2015; Bokhof and Junius-Walker, 2016; Sinnige et al., 2016). Of course, such solutions possess some disadvantages also: they produce dozens of alerts, of which some are of low clinical usefulness, and therefore, subject to overriding (Knight et al., 2019).

Unfortunately, even the availability of such enablers does not guarantee frequent implementation of polypharmacy management mechanisms. A good illustration of the problem is the case of the German FORTA (“Fit fOR The Aged”) guidelines. Originally released in Germany in 2008 as a tool for aiding physicians in screening for unnecessary, inappropriate or harmful medications and drug omissions in older patients in an everyday clinical setting (Wehling, 2009), it was validated in a clinical trial (Wehling et al., 2016), and turned into the application (Pazan and Wehling, 2017). However, a study conducted in 2018 in general practitioners in Baden-Württemberg, Germany revealed that out of 872 surveyed GPs, 39 knew the FORTA list, and 15 declared to use the FORTA App only (Meyer and Wehling, 2020).

This scoping review possesses several limitations. First of all, it was limited to English language publications, and thus, articles published in other languages were excluded. Moreover, among a number of approaches available for polypharmacy management, we were not able to prioritise one over the other, due to the lack of objective benchmarking criteria. Nevertheless, we believe that comprehensive review of available methods provided in this paper will help interested stakeholders make their own choices, and thus, meet the aim of this exercise.

## Conclusion

This scoping review showed a variety of approaches being suggested for and/or employed for the management of polypharmacy in the older adults. These approaches vary in their replicability, complexity, and applicability. The most often recommended ones were various types of drug reviews, guided by either implicit or explicit criteria. Of these, implicit criteria based approaches are used infrequently due to their subjectivity, and limited practical implementability. To the contrary, most of the reviewed publications advocated the use of explicit criteria-based approaches. However, their practical applicability is somehow limited due to very long lists of potentially inappropriate medications covered. To overcome this, that sort of criteria are often embedded in clinical decision support systems.

Our results show that currently, no gold standard exists for polypharmacy management in older adults, and various approaches are used in parallel. Depending on the purpose of drug review, its settings, and available time, the users are free to employ one of existing interventions and/or tools. For practical purposes, employing a drug review based on one of the available explicit criteria seem to be the best choice. Having in mind that in general, polypharmacy management in the older adults is underused, both individual stakeholders, as well as policymakers should strengthen their efforts to promote these activities more strongly.

## Data Availability

The original contributions presented in the study are included in the article/Supplementary Material, further inquiries can be directed to the corresponding author.

## References

[B1] Austad B. Hetlevik I. Mjølstad B. P. Helvik A. S. (2016). Applying Clinical Guidelines in General Practice: a Qualitative Study of Potential Complications. BMC Fam. Pract. 17, 92. 10.1186/s12875-016-0490-3 27449959 PMC4957916

[B2] Barnett K. Mercer S. W. Norbury M. Watt G. Wyke S. Guthrie B. (2012). Epidemiology of Multimorbidity and Implications for Health Care, Research, and Medical Education: a Cross-Sectional Study. Lancet 380 (9836), 37–43. 10.1016/S0140-6736(12)60240-2 22579043

[B3] Beers M. H. (1997). Explicit Criteria for Determining Potentially Inappropriate Medication Use by the Elderly. An Update. Arch. Intern. Med. 157 (14), 1531–1536. 10.1001/archinte.1997.00440350031003 9236554

[B4] Bergert F. W. Braun M. Ehrenthal K. Feßler J. Gross J. Hüttner U. (2014). Recommendations for Treating Adult and Geriatric Patients on Multimedication. Int. J. Clin. Pharmacol. Ther. 52 (Suppl. 1), 1–64. 10.5414/cpp52s001 25310921

[B5] Bokhof B. Junius-Walker U. (2016). Reducing Polypharmacy from the Perspectives of General Practitioners and Older Patients: A Synthesis of Qualitative Studies. Drugs Aging 33 (4), 249–266. 10.1007/s40266-016-0354-5 26915076

[B6] Boyd C. M. Darer J. Boult C. Fried L. P. Boult L. Wu A. W. (2005). Clinical Practice Guidelines and Quality of Care for Older Patients with Multiple Comorbid Diseases: Implications for Pay for Performance. JAMA 294 (6), 716–724. 10.1001/jama.294.6.716 16091574

[B7] Brown J. D. Hutchison L. C. Li C. Painter J. T. Martin B. C. (2016). Predictive Validity of the Beers and Screening Tool of Older Persons' Potentially Inappropriate Prescriptions (STOPP) Criteria to Detect Adverse Drug Events, Hospitalizations, and Emergency Department Visits in the United States. J. Am. Geriatr. Soc. 64 (1), 22–30. 10.1111/jgs.13884 26782849 PMC5287350

[B8] Bulajeva A. Labberton L. Leikola S. Pohjanoksa-Mäntylä M. Geurts M. M. de Gier J. J. (2014). Medication Review Practices in European Countries. Res. Soc. Adm Pharm 10 (5), 731–740. 10.1016/j.sapharm.2014.02.005 24661800

[B9] By the 2019 American Geriatrics Society Beers Criteria® Update Expert Panel (2019). American Geriatrics Society 2019 Updated AGS Beers Criteria® for Potentially Inappropriate Medication Use in Older Adults. J. Am. Geriatr. Soc. 67 (4), 674–694. 10.1111/jgs.15767 30693946

[B10] Cadogan C. A. Ryan C. Francis J. J. Gormley G. J. Passmore P. Kerse N. (2016). Development of an Intervention to Improve Appropriate Polypharmacy in Older People in Primary Care Using a Theory-Based Method. BMC Health Serv. Res. 16 (1), 661. 10.1186/s12913-016-1907-3 27852287 PMC5112618

[B11] Cadogan C. A. Ryan C. Francis J. J. Gormley G. J. Passmore P. Kerse N. (2015). Improving Appropriate Polypharmacy for Older People in Primary Care: Selecting Components of an Evidence-Based Intervention to Target Prescribing and Dispensing. Implement Sci. 10, 161. 10.1186/s13012-015-0349-3 26573745 PMC4647274

[B12] Cadogan C. A. Ryan C. Gormley G. J. Francis J. J. Passmore P. Kerse N. (2017). A Feasibility Study of a Theory-Based Intervention to Improve Appropriate Polypharmacy for Older People in Primary Care. Pilot Feasibility Stud. 4, 23. 10.1186/s40814-017-0166-3.eCollection.2018 28748106 PMC5520366

[B13] Chau S. H. Jansen A. P. van de Ven P. M. Hoogland P. Elders P. J. Hugtenburg J. G. (2016). Clinical Medication Reviews in Elderly Patients with Polypharmacy: a Cross-Sectional Study on Drug-Related Problems in the Netherlands. Int. J. Clin. Pharm. 38 (1), 46–53. 10.1007/s11096-015-0199-8 26597955 PMC4733134

[B14] Clyne B. Bradley M. C. Hughes C. Fahey T. Lapane K. L. (2012). Electronic Prescribing and Other Forms of Technology to Reduce Inappropriate Medication Use and Polypharmacy in Older People: a Review of Current Evidence. Clin. Geriatr. Med. 28 (2), 301–322. 10.1016/j.cger.2012.01.009 22500545

[B15] Clyne B. Cooper J. A. Hughes C. M. Fahey T. Smith S. M. (2016). 'Potentially Inappropriate or Specifically Appropriate?' Qualitative Evaluation of General Practitioners Views on Prescribing, Polypharmacy and Potentially Inappropriate Prescribing in Older People. BMC Fam. Pract. 17 (1), 109. 10.1186/s12875-016-0507-y 27515854 PMC4982127

[B16] Clyne W. Blenkinsopp A. Seal R. (2008). A Guide to Medication Review National Prescribing Centre. UK: NHS. Available at; http://www.cff.org.br/userfiles/52%20-%20CLYNE%20W%20A%20guide%20to%20medication%20review%202008.pdf .

[B17] Cooper J. A. Cadogan C. A. Patterson S. M. Kerse N. Bradley M. C. Ryan C. (2015). Interventions to Improve the Appropriate Use of Polypharmacy in Older People: a Cochrane Systematic Review. BMJ Open 5 (12), e009235. 10.1136/bmjopen-2015-009235 PMC4679890 26656020

[B18] Croke L. (2020). Beers Criteria for Inappropriate Medication Use in Older Patients: An Update from the AGS. Am. Fam. Physician 101 (1), 56–57. 31894929

[B19] Doan J. Zakrzewski-Jakubiak H. Roy J. Turgeon J. Tannenbaum C. (2013). Prevalence and Risk of Potential Cytochrome P450-Mediated Drug-Drug Interactions in Older Hospitalized Patients with Polypharmacy. Ann. Pharmacother. 47 (3), 324–332. 10.1345/aph.1R621 23482734

[B20] Drenth-van Maanen A. C. van Marum R. J. Knol W. van der Linden C. M. Jansen P. A. (2009). Prescribing Optimization Method for Improving Prescribing in Elderly Patients Receiving Polypharmacy: Results of Application to Case Histories by General Practitioners. Drugs Aging 26 (8), 687–701. 10.2165/11316400-000000000-00000 19685934

[B21] Dunning T. (2017). Medicines and Older People: Polypharmacy, Adherence and Safety. Int. Diabetes Nurs. 14 (1), 10–15. 10.1080/20573316.2017.1352142

[B22] Eurostat (2015). People in the EU: Who Are We and How Do We Live? Luxembourg, Publications Office of the European Union.

[B23] Eyigor S. Kutsal Y. G. (2012). Polypharmacy in the Elderly: To Prescribe, or Not Prescribe "that Is the Question". Turk Geriatri Dergisi 15 (4), 445–454.

[B24] Farmer C. Fenu E. O'Flynn N. Guthrie B. (2016). Clinical Assessment and Management of Multimorbidity: Summary of NICE Guidance. BMJ 354, i4843. 10.1136/bmj.i4843 27655884

[B25] Franco J. V. A. Terrasa S. A. Kopitowski K. S. (2017). Medication Discrepancies and Potentially Inadequate Prescriptions in Elderly Adults with Polypharmacy in Ambulatory Care. J. Fam. Med Prim Care 6 (1), 78–82. 10.4103/2249-4863.214962 PMC5629905 29026754

[B26] Fried T. R. O'Leary J. Towle V. Goldstein M. K. Trentalange M. Martin D. K. (2014). Health Outcomes Associated with Polypharmacy in Community-Dwelling Older Adults: a Systematic Review. J. Am. Geriatr. Soc. 62 (12), 2261–2272. 10.1111/jgs.13153 25516023 PMC4270076

[B27] Garpestad E. Devlin J. W. (2017). Polypharmacy and Delirium in Critically Ill Older Adults: Recognition and Prevention. Clin. Geriatr. Med. 33 (2), 189–203. 10.1016/j.cger.2017.01.002 28364991

[B28] Guthrie B. Makubate B. Hernandez-Santiago V. Dreischulte T. (2015). The Rising Tide of Polypharmacy and Drug-Drug Interactions: Population Database Analysis 1995-2010. BMC Med. 13, 74. 10.1186/s12916-015-0322-7 25889849 PMC4417329

[B29] Hamilton H. Gallagher P. Ryan C. Byrne S. O'Mahony D. (2011). Potentially Inappropriate Medications Defined by STOPP Criteria and the Risk of Adverse Drug Events in Older Hospitalized Patients. Arch. Intern. Med. 171 (11), 1013–1019. 10.1001/archinternmed.2011.215 21670370

[B30] Hanlon J. T. Schmader K. E. Samsa G. P. Weinberger M. Uttech K. M. Lewis I. K. (1992). A Method for Assessing Drug Therapy Appropriateness. J. Clin. Epidemiol. 45 (10), 1045–1051. 10.1016/0895-4356(92)90144-c 1474400

[B31] Hanlon J. T. Schmader K. E. (2013). The Medication Appropriateness index at 20: where it Started, where it Has Been, and where it May Be Going. Drugs Aging 30 (11), 893–900. 10.1007/s40266-013-0118-4 24062215 PMC3831621

[B32] Haque R. (2009). ARMOR: a Tool to Evaluate Polypharmacy in Elderly Persons. Ann. Long-term Care 17 (6), 26–30.

[B33] Harugeri A. Joseph J. Parthasarathi G. Ramesh M. Guido S. (2010). Prescribing Patterns and Predictors of High-Level Polypharmacy in the Elderly Population: A Prospective Surveillance Study from Two Teaching Hospitals in India. Am. J. Geriatr. Pharmacother. 8 (3), 271–280. 10.1016/j.amjopharm.2010.06.004 20624616

[B34] Heaton J. Britten N. Krska J. Reeve J. (2017). Person-centred Medicines Optimisation Policy in England: an Agenda for Research on Polypharmacy. Prim. Health Care Res. Dev. 18 (1), 24–34. 10.1017/S1463423616000207 27306579

[B35] Hughes C. M. Cadogan C. A. Patton D. Ryan C. A. (2016). Pharmaceutical Strategies towards Optimising Polypharmacy in Older People. Int. J. Pharm. 512 (2), 360–365. 10.1016/j.ijpharm.2016.02.035 26921516

[B36] Hukins D. Macleod U. Boland J. W. (2019). Identifying Potentially Inappropriate Prescribing in Older People with Dementia: a Systematic Review. Eur. J. Clin. Pharmacol. 75 (4), 467–481. 10.1007/s00228-018-02612-x 30610274

[B37] Jódar-Sánchez F. Malet-Larrea A. Martín J. J. García-Mochón L. López Del Amo M. P. Martínez-Martínez F. (2015). Cost-utility Analysis of a Medication Review with Follow-Up Service for Older Adults with Polypharmacy in Community Pharmacies in Spain: the conSIGUE Program. Pharmacoeconomics 33 (6), 599–610. 10.1007/s40273-015-0270-2 25774017

[B38] Jokanovic N. Wang K. N. Dooley M. J. Lalic S. Tan E. C. Kirkpatrick C. M. (2017). Prioritizing Interventions to Manage Polypharmacy in Australian Aged Care Facilities. Res. Soc. Adm Pharm 13 (3), 564–574. 10.1016/j.sapharm.2016.06.003 27374998

[B39] Kann I. C. Lundqvist C. Lurås H. (2015). Polypharmacy Among the Elderly in a List-Patient System. Drugs Real World Outcomes 2 (3), 193–198. 10.1007/s40801-015-0036-3 27747573 PMC4883218

[B40] Kardas P. Urbański F. Lichwierowicz A. Chudzyńska E. Kardas G. Czech M. (2021). Prevalence and Age Structure of Polypharmacy in Poland: Results of the Analysis of the National Real-World Database of 38 Million Citizens. Front. Pharmacol. 12, 655364. 10.3389/fphar.2021.655364 33935769 PMC8082447

[B41] Kaufman G. (2011). Polypharmacy in Older Adults. Nurs. Stand. 25 (38), 49–58. 10.7748/ns2011.05.25.38.49.c8533 21706978

[B42] Kaufman G. (2017). Polypharmacy and Older People. Nurse Prescribing 15 (3), 140–145. 10.12968/npre.2017.15.3.140

[B43] Kaufmann C. P. Tremp R. Hersberger K. E. Lampert M. L. (2014). Inappropriate Prescribing: a Systematic Overview of Published Assessment Tools. Eur. J. Clin. Pharmacol. 70 (1), 1–11. 10.1007/s00228-013-1575-8 24019054

[B44] Kim J. Parish A. L. (2017). Polypharmacy and Medication Management in Older Adults. Nurs. Clin. North. Am. 52 (3), 457–468. 10.1016/j.cnur.2017.04.007 28779826

[B45] Knight A. M. Maygers J. Foltz K. A. John I. S. Yeh H. C. Brotman D. J. (2019). The Effect of Eliminating Intermediate Severity Drug-Drug Interaction Alerts on Overall Medication Alert Burden and Acceptance Rate. Appl. Clin. Inform. 10 (5), 927–934. 10.1055/s-0039-3400447 31801174 PMC6892645

[B46] Kojima G. Bell C. L. Tamura B. Davis J. Inaba M. Lorenzo P. (2014). Combining Quality Improvement and Geriatrics Training: the Nursing home Polypharmacy Outcomes Project. Gerontol. Geriatr. Educ. 35 (4), 395–408. 10.1080/02701960.2014.907159 24829040 PMC4190157

[B47] Komagamine J. Hagane K. (2017). Intervention to Improve the Appropriate Use of Polypharmacy for Older Patients with Hip Fractures: an Observational Study. BMC Geriatr. 17 (1), 288. 10.1186/s12877-017-0681-3 29246247 PMC5732518

[B48] Laroche M. L. Charmes J. P. Nouaille Y. Picard N. Merle L. (2007). Is Inappropriate Medication Use a Major Cause of Adverse Drug Reactions in the Elderly? Br. J. Clin. Pharmacol. 63 (2), 177–186. 10.1111/j.1365-2125.2006.02831.x 17166186 PMC2000580

[B49] Levy H. B. (2017). Polypharmacy Reduction Strategies: Tips on Incorporating American Geriatrics Society Beers and Screening Tool of Older People's Prescriptions Criteria. Clin. Geriatr. Med. 33 (2), 177–187. 10.1016/j.cger.2017.01.007 28364990

[B51] Lin H.-W. Lin C.-H. Chang C.-K. Chou C.-Y. Yu I.-W. Lin C.-C. (2018). Economic Outcomes of Pharmacist-Physician Medication Therapy Management for Polypharmacy Elderly: A Prospective, Randomized, Controlled Trial. J. Formos. Med. Assoc. 117 (17), 235–243. 10.1016/j.jfma.2017.04.017 28549592

[B52] Maher R. L. Hanlon J. Hajjar E. R. (2014). Clinical Consequences of Polypharmacy in Elderly. Expert Opin. Drug Saf. 13 (1), 57–65. 10.1517/14740338.2013.827660 24073682 PMC3864987

[B53] Mair A. Wilson M. Dreischulte T. (2019). The Polypharmacy Programme in Scotland: Realistic Prescribing. Prescriber 30, 10–16. 08. 10.1002/psb.1779

[B54] Malet-Larrea A. Goyenechea E. Gastelurrutia M. A. Calvo B. García-Cárdenas V. Cabases J. M. (2017). Cost Analysis and Cost-Benefit Analysis of a Medication Review with Follow-Up Service in Aged Polypharmacy Patients. Eur. J. Health Econ. 18 (9), 1069–1078. 10.1007/s10198-016-0853-7 27913940

[B55] Mansur N. Weiss A. Beloosesky Y. (2012). Looking beyond Polypharmacy: Quantification of Medication Regimen Complexity in the Elderly. Am. J. Geriatr. Pharmacother. 10 (4), 223–229. 10.1016/j.amjopharm.2012.06.002 22749668

[B56] Martin-Khan M. G. Edwards H. Wootton R. Varghese P. Lim K. Darzins P. (2016). Web-based (Online) Comprehensive Geriatric Assessment Is More Time Efficient, and as Reliable, as reading Patient Medical Records and Conducting Traditional in Person Consultations. J. Telemed. Telecare 22 (8), 478–482. 10.1177/1357633X16674088 27799451

[B57] Masnoon N. Shakib S. Kalisch-Ellett L. Caughey G. E. (2017). What Is Polypharmacy? A Systematic Review of Definitions. BMC Geriatr. 17 (1), 230. 10.1186/s12877-017-0621-2 29017448 PMC5635569

[B58] May C. Montori V. M. Mair F. S. (2009). We Need Minimally Disruptive Medicine. BMJ 339, b2803. 10.1136/bmj.b2803 19671932

[B59] Mc Namara K. P. Breken B. D. Alzubaidi H. T. Bell J. S. Dunbar J. A. Walker C. (2017). Health Professional Perspectives on the Management of Multimorbidity and Polypharmacy for Older Patients in Australia. Age Ageing 46 (2), 291–299. 10.1093/ageing/afw200 27836856

[B60] McNicholl I. R. Gandhi M. Hare C. B. Greene M. Pierluissi E. (2017). A Pharmacist-Led Program to Evaluate and Reduce Polypharmacy and Potentially Inappropriate Prescribing in Older HIV-Positive Patients. Pharmacotherapy 37 (12), 1498–1506. 10.1002/phar.2043 29023938

[B61] Meyer L. Wehling M. (2020). Knowledge on and Use of the FORTA ("Fit fOR the Aged")-List and the FORTA App by General Practitioners in Baden-Württemberg, Germany. Eur. Geriatr. Med. 11 (3), 499–503. 10.1007/s41999-020-00311-4 32297266 PMC8270821

[B62] Midão L. Giardini A. Menditto E. Kardas P. Costa E. (2018). Polypharmacy Prevalence Among Older Adults Based on the Survey of Health, Ageing and Retirement in Europe. Arch. Gerontol. Geriatr. 78, 213–220. 10.1016/j.archger.2018.06.018 30015057

[B63] Moher D. Liberati A. Tetzlaff J. Altman D. G. Prisma Group (2009). Preferred Reporting Items for Systematic Reviews and Meta-Analyses: the PRISMA Statement. BMJ 339 (4), b2535–W64. 10.1136/bmj.b2535 19622551 PMC2714657

[B64] Monteiro L. Maricoto T. Solha I. Ribeiro-Vaz I. Martins C. Monteiro-Soares M. (20192019). Reducing Potentially Inappropriate Prescriptions for Older Patients Using Computerized Decision Support Tools: Systematic Review. J. Med. Internet Res. 21 (11), e15385. 10.2196/15385 PMC6883366 31724956

[B65] Montori V. M. Brito J. P. Murad M. H. (2013). The Optimal Practice of Evidence-Based Medicine: Incorporating Patient Preferences in Practice Guidelines. JAMA 310 (23), 2503–2504. 10.1001/jama.2013.281422 24165826

[B66] Morin L. Johnell K. Laroche M. L. Fastbom J. Wastesson J. W. (2018). The Epidemiology of Polypharmacy in Older Adults: Register-Based Prospective Cohort Study. Clin. Epidemiol. 10, 289–298. 10.2147/CLEP.S153458 29559811 PMC5856059

[B68] National Guideline Centre. (2016) Multimorbidity: Assessment, Prioritisation and Management of Care for People with Commonly Occurring Multimorbidity. NICE Guideline, London, UK. Available at: nice.org.uk/guidance/ng56 . 27683922

[B69] National Institute for Health and Clinical Excellence. (2015) Medicines management in care homes. London. Published: 25 March 2015, Available at: https://www.nice.org.uk/guidance/qs85/chapter/quality-statement-5-medication-reviews .

[B70] Nobili A. Garattini S. Mannucci P. M. (2011). Multiple Diseases and Polypharmacy in the Elderly: Challenges for the Internist of the Third Millennium. J. Comorb 1, 28–44. 10.15256/joc.2011.1.4 29090134 PMC5556419

[B71] O’Mahony D. O'Sullivan D. Byrne S. O'Connor M. N. Ryan C. Gallagher P. (2015). STOPP/START Criteria for Potentially Inappropriate Prescribing in Older People: Version 2. Age Ageing 44 (2), 213–218. 10.1093/ageing/afu145 25324330 PMC4339726

[B72] O’Mahony D. (2019). STOPP/START Criteria for Potentially Inappropriate Medications/potential Prescribing Omissions in Older People: Origin and Progress. Expert Rev. Clin. Pharmacol. 13, 15–22. 10.1080/17512433.2020.1697676 31790317

[B73] Onder G. Landi F. Liperoti R. Fialova D. Gambassi G. Bernabei R. (2005). Impact of Inappropriate Drug Use Among Hospitalized Older Adults. Eur. J. Clin. Pharmacol. 61 (5–6), 453–459. 10.1007/s00228-005-0928-3 15912391

[B74] Orimo H. (2006). Reviewing the Definition of Elderly. Nihon Ronen Igakkai Zasshi 43 (1), 27–34. 10.3143/geriatrics.43.27 16521795

[B75] Page A. T. Etherton-Beer C. D. Clifford R. M. Burrows S. Eames M. Potter K. (2016). Deprescribing in Frail Older People--Do Doctors and Pharmacists Agree? Res. Soc. Adm Pharm 12 (3), 438–449. 10.1016/j.sapharm.2015.08.011 26453002

[B76] Parekh N. Ali K. Davies J. G. Rajkumar C. (2019). Do the 2015 Beers Criteria Predict Medication-Related Harm in Older Adults? Analysis from a Multicentre Prospective Study in the United Kingdom. Pharmacoepidemiol. Drug Saf. 28 (11), 1464–1469. 10.1002/pds.4849 31338909

[B78] Patterson S. M. Cadogan C. A. Kerse N. Cardwell C. R. Bradley M. C. Ryan C. (2012). Interventions to Improve the Appropriate Use of Polypharmacy for Older People. Cochrane Database Syst. Rev. (5), CD008165. 10.1002/14651858.CD008165.pub3 22592727

[B79] Patterson S. M. Cadogan C. A. Kerse N. Cardwell C. R. Bradley M. C. Ryan C. (2014). Interventions to Improve the Appropriate Use of Polypharmacy for Older People. Cochrane Database Syst. Rev. (10), CD008165. 10.1002/14651858.CD008165.pub3 25288041

[B80] Patton D. E. Hughes C. M. Cadogan C. A. Ryan C. A. (2017). Theory-Based Interventions to Improve Medication Adherence in Older Adults Prescribed Polypharmacy: A Systematic Review. Drugs Aging 34 (2), 97–113. 10.1007/s40266-016-0426-6 28025725 PMC5290062

[B81] Payne R. A. Avery A. J. (2011). Polypharmacy: One of the Greatest Prescribing Challenges in General Practice. Br. J. Gen. Pract. 61, 83–84. 10.3399/bjgp11X556146 21276330 PMC3026143

[B82] Pazan F. Wehling M. (2017). The FORTA (Fit fOR the Aged) App as a Clinical Tool to Optimize Complex Medications in Older People. J. Am. Med. Dir. Assoc. 18 (10), 893. 10.1016/j.jamda.2017.06.031 28780394

[B83] Pereira K. G. Peres M. A. Iop D. Boing A. C. Boing A. F. Aziz M. (2017). Polypharmacy Among the Elderly: a Population-Based Study. Rev. Bras Epidemiol. 20 (2), 335–344. 10.1590/1980-5497201700020013 28832855

[B84] Planton J. Edlund B. J. (2010). Strategies for Reducing Polypharmacy in Older Adults. J. Gerontol. Nurs. 36 (1), 8–12. 10.3928/00989134-20091204-03 20047247

[B85] Quinn K. J. Shah N. H. (2017). A Dataset Quantifying Polypharmacy in the United States. Sci. Data 4, 170167. 10.1038/sdata.2017.167 29087369 PMC5663207

[B86] Rankin A. Cadogan C. A. Patterson S. M. Kerse N. Cardwell C. R. Bradley M. C. (2018). Interventions to Improve the Appropriate Use of Polypharmacy for Older People. Cochrane Database Syst. Rev. 9, CD008165. 10.1002/14651858.CD008165.pub4 30175841 PMC6513645

[B87] Reeve E. Gnjidic D. Long J. Hilmer S. (2015). A Systematic Review of the Emerging Definition of “deprescribing” with Network Analysis: Implications for Future Research and Clinical Practicefinition of “deprescribing” with Network Analysis: Implications for Future Research and Clinical Practice. Br. J. Clin. Pharmacol. 80 (6), 1254–1268. 10.1111/bcp.12732 27006985 PMC4693477

[B88] Rodrigues M. C. Oliveira Cd. (2016). Drug-drug Interactions and Adverse Drug Reactions in Polypharmacy Among Older Adults: an Integrative Review. Rev. Lat Am. Enfermagem 24, e2800. 10.1590/1518-8345.1316.2800 27598380 PMC5016009

[B89] Sabzwari S. R. Qidwai W. Bhanji S. (2013). Polypharmacy in Elderly: a Cautious Trail to Tread. J. Pak Med. Assoc. 63 (5), 624–627. 23757993

[B90] Salisbury C. Johnson L. Purdy S. Valderas J. M. Montgomery A. A. (2011). Epidemiology and Impact of Multimorbidity in Primary Care: a Retrospective Cohort Study. Br. J. Gen. Pract. 61 (582), e12–21. 10.3399/bjgp11X548929 21401985 PMC3020068

[B91] Schöpf A. C. von Hirschhausen M. Farin E. Maun A. (2017). Elderly Patients' and GPs' Perspectives of Patient-GP Communication Concerning Polypharmacy: a Qualitative Interview Study. Prim. Health Care Res. Dev. 19, 355–364. 10.1017/S1463423617000883 29277160 PMC6452947

[B92] Scottish Government Polypharmacy Model of Care Group (2018). Polypharmacy Guidance, Realistic Prescribing. 3rd Edition. Scottish Government. Available at: https://www.therapeutics.scot.nhs.uk/wp-content/uploads/2018/04/Polypharmacy-Guidance-2018.pdf .

[B93] Sergi G. De Rui M. Sarti S. Manzato E. (2011). Polypharmacy in the Elderly: Can Comprehensive Geriatric Assessment Reduce Inappropriate Medication Use? Drugs Aging 28 (7), 509–518. 10.2165/11592010-000000000-00000 21721596

[B94] Sharma M. Loh K. P. Nightingale G. Mohile S. G. Holmes H. M. (2016). Polypharmacy and Potentially Inappropriate Medication Use in Geriatric Oncology. J. Geriatr. Oncol. 7 (5), 346–353. 10.1016/j.jgo.2016.07.010 27498305 PMC5037024

[B95] Shaw J. Seal R. (2015). Pilling M on Behalf of the Task Force on Medicines Partnership and the National Collaborative Medicines Management Services Programme. Room for Review. A Guide to Medication Review: the Agenda for Patients, Practitioners and Managers. National Prescribing Centre. Available at: http://www.npc.co.uk/med_partnership/medication-review/room-for-review/downloads.html .

[B96] Sinnige J. Korevaar J. C. van Lieshout J. Westert G. P. Schellevis F. G. Braspenning J. C. (2016). Medication Management Strategy for Older People with Polypharmacy in General Practice: a Qualitative Study on Prescribing Behaviour in Primary Care. Br. J. Gen. Pract. 66 (649), e540–51. 10.3399/bjgp16X685681 27266862 PMC4979928

[B97] Stewart D. Gibson-Smith K. MacLure K. Mair A. Alonso A. Codina C. (2017). A Modified Delphi Study to Determine the Level of Consensus across the European Union on the Structures, Processes and Desired Outcomes of the Management of Polypharmacy in Older People. PLoS One 12 (11), e0188348. 10.1371/journal.pone.0188348.eCollection.2017 29155870 PMC5695766

[B98] Stewart D. Mair A. Wilson M. Kardas P. Lewek P. Alonso A. (2017). Guidance to Manage Inappropriate Polypharmacy in Older People: Systematic Review and Future Developments. Expert Opin. Drug Saf. 16 (2), 203–213. 10.1080/14740338.2017.1265503 27885844

[B99] The Scottish Government Polypharmacy. (2018) The Scottish Government Polypharmacy: Manage Medicines. Available at: https://managemeds.scot.nhs.uk/ .

[B100] Tommelein E. Mehuys E. Van Tongelen I. Petrovic M. Somers A. Colin P. (2017). Community Pharmacists' Evaluation of Potentially Inappropriate Prescribing in Older Community-Dwelling Patients with Polypharmacy: Observational Research Based on the GheOP³S Tool. J. Public Health (Oxf) 39 (3), 583–592. 10.1093/pubmed/fdw108 27698269

[B101] Urfer M. Elzi L. Dell-Kuster S. Bassetti S. (2016). Intervention to Improve Appropriate Prescribing and Reduce Polypharmacy in Elderly Patients Admitted to an Internal Medicine Unit. PLoS One 11 (11), e0166359. 10.1371/journal.pone.0166359.eCollection.2016 27902720 PMC5130196

[B102] Van der Linden L. Decoutere L. Decoutere L. Flamaing J. Spriet I. Willems L. (2014). Development and Validation of the RASP List (Rationalization of Home Medication by an Adjusted STOPP List in Older Patients): A Novel Tool in the Management of Geriatric Polypharmacy. Eur. Geriatr. Med. 5 (3), 175–180. 10.1016/j.eurger.2013.12.005

[B103] Verdoorn S. Kwint H. F. Faber A. Gussekloo J. Bouvy M. L. (2015). Majority of Drug-Related Problems Identified during Medication Review Are Not Associated with STOPP/START Criteria. Eur. J. Clin. Pharmacol. 71 (10), 1255–1262. 10.1007/s00228-015-1908-x 26249851 PMC4564444

[B104] Wehling M. Burkhardt H. Kuhn-Thiel A. Pazan F. Throm C. Weiss C. (2016). VALFORTA: a Randomised Trial to Validate the FORTA (Fit fOR the Aged) Classification. Age Ageing 45 (2), 262–267. 10.1093/ageing/afv200 26786346

[B105] Wehling M. (2009). Multimorbidity and Polypharmacy: How to Reduce the Harmful Drug Load and yet Add Needed Drugs in the Elderly? Proposal of a New Drug Classification: Fit for the Aged. J. Am. Geriatr. Soc. 57, 560–561. 10.1111/j.1532-5415.2009.02131.x 19278399

[B106] Wilson M. Mair A. Dreischulte T. Witham M. D. (2015). Prescribing to Fit the Needs of Older People-Tthe NHS Scotland Polypharmacy Guidance, 2nd Edition. J. R. Coll. Physicians Edinb. 45 (2), 108–113. 10.4997/JRCPE.2015.204 26181524

[B107] World Health Organization (2019). Medication Safety in Polypharmacy. Geneva. (WHO/UHC/SDS/2019.11).

[B108] World Health Organization (2008). The World Health Report 2008. Primary Health Care - Now More than Ever. New York: The World Health Organization.

[B109] Yamanouchi Y. Sukegawa T. Inagaki A. Inada T. Yoshio T. Yoshimura R. (2014). Evaluation of the Individual Safe Correction of Antipsychotic Agent Polypharmacy in Japanese Patients with Chronic Schizophrenia: Validation of Safe Corrections for Antipsychotic Polypharmacy and the High-Dose Method. Int. J. Neuropsychopharmacol. 18 (5), pyu016. 10.1093/ijnp/pyu016) 25522380 PMC4376537

[B110] Zelko E. Klemenc-Ketis Z. Tusek-Bunc K. (2016). Medication Adherence in Elderly with Polypharmacy Living at home: a Systematic Review of Existing Studies. Mater. Sociomed 28 (2), 129–132. 10.5455/msm.2016.28.129-132 27147920 PMC4851507

