# Supplementary material for: Polypharmacy Management in the Older Adults: A Scoping Review of Available Interventions
Source: Front Pharmacol. 2021 Nov 26;12:734045. doi: 10.3389/fphar.2021.734045 (PMC8661120; doi:10.3389/fphar.2021.734045)
Supplement: Supplementary file 1 [file Table1.docx]

| (multimedic*[ti] OR “multiple medication"[ti] OR polifarma*[ti] OR polyfarma*[ti] OR polimedicin*[ti] OR polymedicin*[ti] OR polipharma*[ti] OR polypharma*[ti] OR polipragma*[ti] OR polypragma*[ti] OR politerap* [ti] OR polyterap* [ti]) AND (interven*[Tiab] OR strateg*[Tiab] OR guidelin* [tiab]) AND (elder* [ti] OR old* [ti] OR age* [ti] OR geriatric*[ti)  Limiters – published date 01/01/2010 onward; language - English |
| --- |

**Online material 1.** Review search terms, as formatted for PubMed
