# Supplementary material for: Polypharmacy Management in the Older Adults: A Scoping Review of Available Interventions
Source: Front Pharmacol. 2021 Nov 26;12:734045. doi: 10.3389/fphar.2021.734045 (PMC8661120; doi:10.3389/fphar.2021.734045)
Supplement: Supplementary file 2 [file Table2.docx]

| **Reference** | **The country of origin** | **Methodology** | **Disease** | **Intervention type** | **Intervention settings and HCPs involved** | **Study outcome** |
| --- | --- | --- | --- | --- | --- | --- |
| [7] Stewart D et al. (2017) | NA | Systematic review | not specified | Suggested: drug review, using tools to assess potentially inappropriate prescribing | Not stated | NA |
| [23] Levy HB (2017) | NA | Review | not specified | SUGGESTED: Drug list review + identification of potentially inappropriate medications using implicid-based approaches (e.g. ARMOR, POM) or implicid-based approaches (AGS Beers, STOPP) | Not stated | NA |
| [26] Sergi G et al. (2011) | NA | Review | not specified | SUGGESTED: Comprehensive geriatric assessment (CGA) followed by drug review (using tools e.g. Beers, MAI, STOPP/START, GPGP) | interdisciplinary team (comprising nurses, occupation and physical therapists, social workers, general practitioners and geriatricians) | NA |
| [45] Bokhof B et al. (2016) | NA | systematic review | multimorbidity | SUGGEDTED INTERVENTIONS: for patients: awareness of their complex multiple medication regimens and a better position to receive sufficient information about their drugs. Enhanced health literacy and partnerlike relationship; for GPs: medical IT support dealing with interactions and dosage adjustments, evidence-based (de)prescribing procedures, a trusting relationship and shared decision making approached on an equal basis; for health system: protected consultation time for medication reviews, clear prescribing roles and responsibilities for interface management | GPs in primary care | 11 key concepts of GPs and patients on reducing polypharmacy; A patient-centered consultation style and a certain level of patient health literacy are key heuristics facing this issue. |
| [46] Cadogan CA et al. (2015) | UK | Original article (Focus group) | not specified | NA | NA | 8 key domains were identified as components of an evidence-based intervention to target prescribing and dispensing in elderly: ‘Skills’, ‘Beliefs about capabilities’, ‘Beliefs about consequences’, ‘Environmental context and resources’, ‘Memory, attention and decision processes’, ‘Social/professional role and identity’, ‘Social influences’ and ‘Behavioural regulation’. |
| [47] Cadogan CA et al. (2016) | UK | Original article (theoretical study) | not specified | INTERVENTION: short online video (or series of videos) demonstrating how GPs can prescribe appropriate polypharmacy during a typical consultation with an older patient | GP's and pharmacists in primary care | Three draft interventions comprising selected behaviour change techniques were developed out of which the GP-targeted intervention was selected for feasibility testing |
| [48] Cadogan CA et al. (2018) | UK | Original article (A feasibility study of theory-based intervention) | not specified | Suggested: appropriate prescribing and medication review. | GPs in primary care | The intervention was considered usable and acceptable by GPs. Patients welcomed the opportunity to have their medications reviewed. |
| [49] Chau SH et al. (2016) | Denmark | Original article (cross-sectional study) | not specified | INTERVENTIONS: Stopping to use a drug was suggested most frequently (19.6 %), second most suggested was monitoring of the patient (18.4 %), e.g. measuring of the blood pressure or performing a blood test. | Pharmacists in community pharmacies | Overtreatment (25 %) and undertreatment were the most frequently identified drug related problems. Drugs for peptic ulcer and gastro-oesophageal reflux disease, antithrombotic agents and lipid modifying agents were most frequently involved in overtreatment. Nearly half (46.2 %) of all pharmacist-proposed interventions. STOPP/START criteria may be useful Using Amsterdam Tool for additional support of the adressing DPRs may be useful. |
| [50] Clyne B et al. (2012) | NA | Review | not specified | Suggested: E-prescribing and other forms of technology, such as CDSS, to reduce inappropriate prescribing | Not stated | NA |
| [51] Clyne B et al. (2016) | Ireland | Original article (Qualitative study) | NA | Not defined | GPs in primary and secondary care | NA |
| [52] Cooper JA et al. (2015) | NA | Systematic review (12 studies included) | >= 1 long term condition | SUGGESTED: Pharmaceutical care-based intervention based on drug review, using validated assessment criteria to give recommendations on improving the appropriateness of prescribing (11 studies); computerised decision support (CDS) provided to GPs (1 study) | Pharmacists, GPs | Interventions demonstrated improvements in appropriate polypharmacy based on reductions in inappropriate prescribing. However, it remains unclear if interventions resulted in clinically significant improvements . |
| [53] Doan J, et al. (2013) | Canada | Original article (Prospective cohort study) | not specified | TOOL - SOFTWARE: Analysis of the patients medication profiles with a new multidrug cytochrome-specific software program. The prevalence of potential CYP-mediated DDIs was determined, with the probability calculated as a function of the number of medications dispensed using multivariate Poisson regression adjusted for age and sex. | Pharmacists in community hospital | The strategy behind the multidrug potential CYP-mediated DDI analysis software is to provide a clinical support tool to inform clinical judgment about risk reduction management for patients with polypharmacy. |
| [54] Dunning K (2017) | Australia | Review Article | Diabetes | RECOMMENDATIONS: Strategies to improve medicine safety+ compliance and adherence+ strategies help reduce medicine-related errors and adverse events: 1. Information safely and effectively; 2. Measuring medication adherence; 3. Medicine self-managerment and important role of personalised care ; 4. A good relationship between the patients and the HP; 5. proactively monitor and identify older people with diabetes at risk of non-adherence, errors and AEs; 6. Carers are also an important source of information 7. Using non-medicine options where possible and stopping unnecessary medicines to reduce polypharmacy. 8. Undertaking regular comprehensive medicine reviews | GPs, governance and policy | N/A |
| [55] Eyigor S, Kutsal Y.G. (2012) | Turkey | Review Articles | not specified | RECOMMENDATIONS: multidimensional geriatric assessment, golden rules of prescribing, pilot applications include provision of product information in hard copies by companies, on-line access to patients information, alerts through special programs when inappropriate drugs are prescribed, access to these system through a personal digital assistant or a mobile phone, assessment of the effects of accompanying disease, reviewing potential drug-drug interactions, graduate and post-graduate education, multidisciplinary approach, electronic systems, home visits and home monitoring systems | Health professional (physicians, pharmacists, dentists, nurses) | N/A |
| [56] Franco JV et al. (2018) | Argentina | Original article (Cross-sectional study) | not specified | Frequency and type of MD and PIP and association between the number of MD and PIP and other demographic and clinical variables | GPs in ambulatory care in a Private Academic Community Hospital | Proportion of patients with AD and PIP was very high. Number of prescription was strongly associated with number of MD. Interventions should be aimed at reducing the number of PIP to prevent adverse events and improve EMR accuracy by lowering medications discrepancies |
| [57] Garpestad E et al. (2017) | USA | Review | Deliurium in critically ill | Suggestion: Assessment of the relationship between polypharmacy and delirium in critically ill, older adults diuring and after ICU stay (particularly antipsychotics delirium treatment interventions)+ proposal for a strategy on how to recognize and reduce medication - delirium and recomendations on how to reduce or prevent polypharmacy in patients who develops delirium | Clinicians in ICU | N/A |
| [58] A. Harugeri et al. (2010) | India | Original article (A prospective surveillance study) | not specified | INTERVENTION - The campaign for rational drug use in the elderly population should promote the prescribing of atorvastatin, ceftriaxone, and pantoprazole in concordance with their indications for use. Interventions to reduce high-level polypharmacy during stays in tertiary care hospitals should focus on patients who have multiple diagnoses, angina pectoris, and/or an LOS ≥10 days. | clinical pharmacist in teaching hospitals | The majority of the medications frequently prescribed during hospital stays were prescribed as indicated. The medications most commonly prescribed off-label were pantoprazole, ceftriaxone, and atorvastatin. |
| [59] Heaton J et al. (2017) | England | Review | not specified | Discussed: guidelines on medicines optimisation published by the Royal Pharmaceutical Society (RPS), The King’s Fund and National Institute for Health and Social Care Excellence | NA | NA |
| [60] Hughes CM et al.. (2016) | Na | Review | not specified | SUGGESTED: Drug list review + identification of potentially inappropriate medications using screening tools (Beers Criteria, STOPP/START, PRISCUS, NORGEP, LaRoche), using patient-adjusted pharmaceutical formulation, using strategies to improve adherence | Not stated | NA |
| [61] Jokanovic N et al. (2017) | Australia | Original article (focus group) | not specified | INTERVENTION: implementation of a pharmacist-led medication reconciliation service for new residents of residential aged care facilities, Facility-level audit and feedback to staff and health care professionals on high risk medications, Develop ‘deprescribing scripts’ to assist GPs and other clinicians to discuss medication discontinuation | pharmacists, GPs in residential aged care facilities | Six of the 16 potential interventions were prioritized highest for possible implementation in clinical practice. The top interventions were ‘implementation of a pharmacist-led medication reconciliation service for new residents,’ |
| [62] Jódar-Sánchez F et al. (2015) | Spain | cluster RCT | not specified | INTERVENTION: The medication review with follow-up (MRF) | Pharmacists in community pharmacies | The medication review with follow-up (MRF) service is an effective intervention for optimizing prescribed medication and improving quality of life in older adults with polypharmacy in community pharmacies. The results from the cost-utility analysis suggest that the MRF service is cost effective. |
| [63] Kann IC et al. (2015) | Norway | Original article (database study) | not specified | none (intervention IS NOT advised: 'Norwegian list-patient system does not prevent polypharmacy') | regular GPs, another GPs (=non-regular ones), non-GP specialists, hospital doctors | GPs prescribe all the substances that cause polypharmacy in 64 % of the incidents, but the patients’ risk of polypharmacy increases substantially with number of prescribers. Any intervention intended to improve polypharmacy in elderlies must include GPs. |
| [64] Kaufman G (2011) | NA | Review | not specified | Suggested: optimal prescribing and medication review. | Primary care workers | NA |
| [65] Kaufman G (2017) | UK | Review Article | not specified | RECOMMENDATIONS: Seven steps approach to medication review. Five - steps patient-centered deprescribing. | Health professional, physician | N/A |
| [66] Kim J et al. (2017) | NA | Review | not specified | NA | NA | NA |
| [67] Kojima G et al. (2014) | USA | Original article (Prospective) | not specified | Drug list review + identification of potentially inappropriate medications using the Beers Criteria + potential drug-drug interactions and contraindicated medications using Epocrates online drug-drug interaction program | Geriatric Medicine fellows (under supervision of Medical Director and Director of Nursing) in nursing home | Intervention demonstrated decreased potentially inappropriate medications, contraindicated medications, and medication costs. |
| [68] Komagamine J et al. (2017) | Japan | Original article (A retrospective observational study.) | Hip fracture | The intervention consisted of an assessment of the appropriateness of polypharmacy and the de-prescription of any unnecessary medications during the patients’ hospital stay. | Internal medicine physicians, pharmacist in acute care hospital | The intervention was associated with a reduction in potentially inappropriate medications but not an improvement in clinical outcomes. This intervention, which focused only on polypharmacy, may not effectively improve outcomes for elderly patients with hip fractures. |
| [69] Lin HW et al. (2018) | Taiwan | Prospective RCT | not specified | INTERVENTION: Collaborative physician-pharmacist medication therapy management (MTM) program for polypharmacy elderly patients. Patients randomized to MTM program were monitored continuously by a clinical pharmacist. | The physician-pharmacist MTM group team included: 2 geriatricians, 1 cardiologist, 1 nephrologist, 1 clinical pharmacist supervisor in outpatient clinics of teaching hospital | Collaborative physician-pharmacist MTM program targeting polypharmacy geriatric patients was truly cost saving. Non-clinically significant benefits on certain clinical and humanistic outcomes. |
| [70] Malet-Larrea A et al. (2017) | Spain | cluster RCT | not specified | INTERVENTION: Medication review with follow-up (MRF) | Pharmacists in community pharmacies | decrease of uncontrolled health problems in the IG more than 50% (p<.001). The investment needed by pharmacies to provide the MRF service in the conSIGUE Program was €210.8 (SD: 32.8) The QALYs obtained were 0.3721 (0.12) in the IG and 0.3488 (0.15) in the CG (p = 0.002). |
| [71] Mansur N et al. (2012) | Israel | Original article (Cohort study, divided into 2 stages: prospective and retrospective) | not specified | NA | Pharmacist in acute geriatric ward | The MRCI showed satisfactory validity and good evidence of classifying regimen complexity over a simple medication count. The MRCI demonstrated application in clinical research and practice in the elderly. |
| [72] McNicholl IR et al. (2017) | USA | Prospective RCT | HIV | SUGGESTED: Drug review utilizing Beers and STOPP criteria | Clinician + clinical pharmacist in large urban HIV clinic | Pharmacist-led review of medication prescribing using Beers and STOPP criteria revealed a large number of PIP, many amenable to immediate clinical pharmacist intervention. |
| [73] Nobili A et al. (2011) | NA | Review | not specified | Suggested: more rational and conservative drug prescribing, medication review. | Internists | Improve skills for a comprehensive evaluation of each patient, assessing clinical problems and also functional, cognitive, behavioral, and socioeconomic issues |
| [74] Patterson SM et al. (2012) | NA | Systematic review | not specified | INTERVENTIONS: pharmaceutical care, Computerised decision support | pharmacists, physicians in pharmacies | pharmaceutical care appears to improve prescribing for older patients receiving polypharmacy, especially when a multi-disciplinary element is included in the provision of care; Computerised decision support appears to be a helpful intervention for improving appropriate polypharmacy |
| [75] Patterson SM et al. (2014) | NA | Systematic review | not specified | Suggested: Assessment of appropriateness of prescribing measured using validated tools, including the MAI score post intervention (eight studies), Beers criteria (four studies), STOPP criteria (two studies) and START criteria (one study). | prescribers (physicians) and pharmacists. | 12 included studies. One intervention consisted of computerised decision support; 11 complex, multi-faceted pharmaceutical approaches to interventions were provided in a variety of settings. Interventions were delivered by healthcare professionals, such as prescribers and pharmacists. Interventions included in this review resulted in a reduction in inappropriate medication usage. Based on the GRADE approach, the overall quality of evidence for all pooled outcomes ranged from very low to low. Evidence of the effects of interventions on hospital admissions (five studies) and of medication-related problems (six studies) was conflicting. Conclusions It is unclear whether interventions to improve appropriate polypharmacy, such as pharmaceutical care, resulted in clinically significant improvement; however, they appear beneficial in terms of reducing inappropriate prescribing. |
| [76] Patton DE et al. (2017) | NA | systematic review | not specified | none | nurses, researchers, health educators | Theory is rarely used in the development of adherence interventions for older adults prescribed polypharmacy. Details of exactly how theory informs intervention development are often lacking. More adherence interventions with a robust theoretical basis are required. |
| [77] Planton J et al. (2010) | NA | Review | not specified | Suggested: Drug list review; identification of potentially inappropriate medications using the ARMOR and Beers Criteria; avoiding drugs covering side effects of other drugs; avoiding drugs with anticholinergic properties | Not stated | NA |
| [78] Rodrigues MC et al.. (2016) | NA | Integrative review | not specified | NA | NA | NA |
| [79] Sabzwari SR et al. (2013) | Pakistan | Review | not specfied | NA | Primary care workers | NA |
| [80] Schöpf AC, et al. (2017) | Germany | Original article (Qualitative semi - structured interview study) | not specified | Suggested: highlighted patients’ active role in addressing polypharmacy. Thus, interventions which improve patients’ communication skills and address specific issues of polypharmacy, particularly in elderly patients, should be designed. | GPs in primary care | N/A |
| [81] Sharma M et al. (2017) | NA | Review | Geriatric oncology | Suggested: Drug list review; identification of potentially inappropriate medications using the Beers Criteria, STOPP, MAI, or NCCN guifdelines; comprehensive medication assessment (as a part of geriatric assessment); deprescribing | Various; authors suggest utilization of pharmacists as part of healthcare delivery model | NA |
| [82] Sinnige J et al. (2016) | Netherlands | Original article (Qualitative study (two focus groups)) | not specified | Discussed: about extensive and structured collaboration between healthcare professionals (eg. GPs and pharmacists) in order to facilitate decision making support medication management for patients with polypharmacy | GPs in primary care | Suggestion of use decision-making support tools, such as BADRI and any other supported tolls incorporated into GP EMR . |
| [83] Stewart D et al. (2017) | EU member states | Original article (modified Delphi study) | not specified | NA | NA | Consensus was obtained for 27/46 (58.7%) statements in Round 1, with consensus obtained for a further two statements (29/46, 63.0%) in Round 2 and nil in Round 3 |
| [84] Tommelein E et al. (2016) | Belgium | Original article (Prospective observational study) | not specified | SCREENING TOOL: Screening for potentially inappropriate prescribing with the GheOPS tool | Pharmacist and phisician in primary care | Screening with the GheOPS tool revealed a high prevalence of PIP in community-dwelling older polypharmacy patients. A higher number of drugs, female gender, a higher BMI and a poorer functional status are risk factors for a higher PIP prevalence. The usability of the GheOP³S tool is acceptable although digitalization of the tool would improve its feasibility. |
| [85] Urfer M et al. (2016) | Switzerland | Original article (non-RCT interventional study) | not specified | INTERVENTIONS: checklist improving quality of drug prescriptions | Physicians in division of Internal Medicine, University of Teaching Hospotal | The intervention with the checklist was associated with a significant reduction by 22% of the risk of being prescribed 1 potentially inappropriate medications at discharge. A higher number of prescribed drugs at admission and not living at home were independently associated with a reduction 20% of prescribed drugs at discharge |
| [86] Van Der Linden L et al. (2018) | Belgium | Original article (development and validation of new the RASP list) | not specified | SCREENING TOOL: Develop a novel screening instrument (checklist) to systematically identify and subsequently reduce potentially inappropriate medications based on information from STOPP, Renal Drug HandBook or Summary of Product Characteristic. | Pharmacists and geriatricians' only at acute geriatric wards | Development of RASP 2.0 list which is a new screening tool that identifies potentially inappropriate medications in elderlies |
| [87] Von Ferber L. et al. (2014) | Germany | Guidelines | not specified | Discussed: guidelines on medicines optimisation and 8 key following steps were identified as components of prescription process | GPs | N/A |
| [88] Wilson M et al. (2015) | UK | Guidelines | not specified | Drug Review Process according to this Polypharmacy Guidance | physicians and pharmacists | drug review process; focus on the patient as a whole rather as a jigsaw of conditions that the medication review process aims for. |
| [89] Yamanouchi Y, et al. (2015) | Japan | Clinical trial (Randomized open study) | Schizophrenia | Antipsychotic agent dose reduction (1 antipsychotic agents was reduced every week) (the SCAP method) to minimize the risk of adverse effects caused by the abrupt discontinuation of long-term exposure to high doses of antipsychotic agents in schizophrenia treatment | Physiscian in outpatient clinic and hospital | Dose reduction method were observed not to exhibit any significant difference in psychiatric symptoms or nonpsychiatric side effects + application of the SCAP method in clinical practice may positively influence the decision to reduce polypharmacy |
| [90] Zelko E et al. (2016) | Spain, USA, Denmark, Italy | Systematic review | not specified | none (systematic review focused on adherence) | NA | Medication adherence was negatively associated with large caregiver burden, impaired hearing, poor cognition, and greater number of drugs . The adherence was positively associated with the use of over-the counter drugs, in patients with higher educational level, in women, and with higher scores on the executive function test. A systematic review of intervention to improve medication adherence revealed several problems regarding the inconsistent methodology. |
