# Supplementary material for: Polypharmacy Management in the Older Adults: A Scoping Review of Available Interventions
Source: Front Pharmacol. 2021 Nov 26;12:734045. doi: 10.3389/fphar.2021.734045 (PMC8661120; doi:10.3389/fphar.2021.734045)
Supplement: Supplementary file 3 [file Table3.docx]

**Online material 3**. Characteristics of selected studies describing development of new screening tools for polypharmacy.

| Reference | The country of origin | Methodology | Disease | Intervention type | Intervention settings and HCPs involved | Study outcomes |
| --- | --- | --- | --- | --- | --- | --- |
| [86] Van Der Linden L et al. (2018) | Belgium | Original article (development and validation of new the tool - RASP list) | Not specified | Develop a novel screening instrument (checklist) for HCPs to systematically identify and subsequently reduce PIMs based on information from STOPP, Renal Drug HandBook or Summary of Product Characteristic. | Pharmacists and geriatricians in acute geriatric wards | Development of RASP 2.0 list which is a new screening tool that identifies PIMs in elderlies. |
| [84] Tommelein E et al. (2016) | Belgium | Prospective observational study | Not specified | Screening for PIP with the GheOPS tool | Pharmacist and physician in primary care | Screening with the GheOPS tool revealed a high prevalence of PIP in community-dwelling older polypharmacy patients.  The usability of the GheOP³S tool is acceptable although digitalization of the tool would improve its feasibility. |
| [53] Doan J, et al. (2013) | Canada | Prospective cohort study | Not specified | Analysis of the patients medication profiles with a new multidrug cytochrome-specific software program. | Pharmacists at community hospital | The strategy behind the multidrug potential CYP-mediated DDI analysis software is to provide a clinical support tool to inform clinical judgment about risk reduction management for patients with polypharmacy. |

*STOPP - The Screening Tool of Older Person’s Prescriptions; PIMs – Potentially Inappropriate Medications HCPs – Healthcare professionals; PIP – Potentially Inappropriate Prescribing; DDI – drug-drug interaction
