# Supplementary material for: Polypharmacy Management in the Older Adults: A Scoping Review of Available Interventions
Source: Front Pharmacol. 2021 Nov 26;12:734045. doi: 10.3389/fphar.2021.734045 (PMC8661120; doi:10.3389/fphar.2021.734045)
Supplement: Supplementary file 4 [file Table4.docx]

**Online material 4.** Characteristics of identified studies testing various intervention targeting polypharmacy management in elderly

| Reference | The country of origin | Methodology | Disease | Intervention type | Intervention settings and HCP’s | Study outcomes |
| --- | --- | --- | --- | --- | --- | --- |
| [56] Franco JV et al. (2018) | Argentina | Cross-sectional study | not specified | Frequency and type of MD and PIP and association between the number of MD and PIP and other demographic and clinical variables | GPs in ambulatory care in a private academic community hospital | Proportion of patients with AD and PIP was very high. Number of prescription was strongly associated with number of MD. Interventions should be aimed at reducing the number of PIP to prevent adverse events and improve EMR accuracy by lowering medications discrepancies |
| [71] Mansur N et al. (2012) | Israel | Cohort study, divided into 2 stages: prospective and retrospective | Not specified | MRCI | Pharmacists in acute geriatric ward | The MRCI showed satisfactory validity and good evidence of classifying regimen complexity over a simple medication count. The MRCI demonstrated application in clinical research and practice in the elderly. |
| [68] Komagamine J et al. (2017) | Japan | A retrospective observational study. | Hip fracture | The intervention consisted of an assessment of the appropriateness of polypharmacy and the de-prescription of any unnecessary medications during the patients’ hospital stay | Internal medicine physicians, pharmacist in acute care hospital | The intervention was associated with a reduction in PIMs but not an improvement in clinical outcomes. This intervention, which focused only on polypharmacy, may not effectively improve outcomes for elderly patients with hip fractures. |
| [69] Lin HW et al. (2018) | Taiwan | Prospective RCT | not specified | Collaborative physician-pharmacist MTM program for polypharmacy elderly patients. | The MTM group team included: 2 geriatricians, 1 cardiologist, 1 nephrologist, 1 clinical pharmacist supervisor in outpatient clinics of teaching Hospital | Collaborative physician-pharmacist MTM program targeting polypharmacy geriatric patients was truly cost saving. Non-clinically significant benefits on certain clinical and humanistic outcomes |
| [85] Urfer M et al. (2016) | Switzerland | Original article (non-RCT interventional study) | Not specified | 5-point checklist improving quality of drug prescriptions | Physicians at Division of Internal Medicine, university teaching hospital | The intervention with the checklist was associated with a significant reduction by 22% of the risk of being prescribed 1 potentially inappropriate medications at discharge |
| [70] Malet-Larrea A et al. (2017) | Spain | Cluster RCT | Not specified | Medication review with follow-up (MRF) | Pharmacists at community pharmacies | Decrease of uncontrolled health problems in the IG more than 50% (p<.001) |
| [47] Cadogan CA et al. (2016) | Northern Ireland, UK | Original article (theoretical study) | Not specified | Short online video (or series of videos) demonstrating how GPs can prescribe appropriate polypharmacy during a typical consultation with an older patient | GPs and pharmacists in primary care | Three draft interventions comprising selected behaviour change techniques were developed out of which the GP-targeted intervention was selected for feasibility testing. |
| [61] Jokanovic N et al. (2017) | Australia | Original article (focus group) | Not specified | Implementation of a pharmacist-led medication reconciliation service for new residents. Facility-level audit and feedback to staff and health care professionals on high risk medications, Develop ‘deprescribing scripts’ to assist GPs and other clinicians to discuss medication discontinuation | Pharmacists, GPs at residential aged care facilities | Six of the 16 potential interventions were prioritized highest for possible implementation in clinical practice. The top interventions were: ‘implementation of a pharmacist-led medication reconciliation service for new residents,’  ‘conduct facility level audits and feedback to staff and health care professionals,’  ‘develop deprescribing scripts to assist clinician-resident discussion,’ |
| [63] Kann IC et al. (2015) | Norway | Original article (database study) | Not specified | none (intervention IS NOT advised: 'Norwegian list-patient system does not prevent polypharmacy') | regular GPs, another GPs (=non-regular ones), non-GP specialists, hospital doctors | GPs prescribe all the substances that cause polypharmacy in 64 % of the incidents, but the patients’ risk of polypharmacy increases substantially with number of prescribers, odds ratio 2.32 (95 % CI 2.31–2.33). Any intervention intended to improve polypharmacy in elderlies must include GPs. |
| [62] Jódar-Sánchez F et al. (2015) | Spain | Cluster RCT | Not specified | The medication review with follow-up (MRF) | Pharmacists at community pharmacies | Greater reduction of prescribed medicines and hospitalizations in IG, improvement of quality of life in IG, reduction in the mean daily cost of prescribed medication in IG, CG reported a greater consumption of healthcare resources. The MRF service is an effective intervention for optimizing prescribed medication and improving quality of life in older adults with polypharmacy in community pharmacies. The results from the cost-utility analysis suggest that the MRF service is cost effective. |
| [72] McNicholl IR et al. (2017) | USA | Prospective RCT | HIV patients | Drug review utilizing Beers and STOPP criteria | Clinicians with clinical pharmacists at large urban HIV clinic | Pharmacist-led review of medication prescribing using Beers and STOPP criteria revealed a large number of PIP, many amenable to immediate clinical pharmacist intervention. |
| [67] Kojima G et al. (2014) | USA | Prospective study | Not specified | Drug list review identification of PIMs using the Beers Criteria  potential DDIs and contraindicated medications using Epocrates online DDI program | Geriatric Medicine fellows at nursing home | Intervention demonstrated decreased PIMs, contraindicated medications, and medication costs. |

*STOPP - The Screening Tool of Older Person’s Prescriptions; MRCI - Medication Regimen Complexity Index MTM - medication therapy management; PIMs – Potentially Inappropriate Medications; DDIs – drug-drug interactions; PIP – potentially inappropriate prescribing; IG – intervention group; CG – control group;
